# Isotopic evidence for pallasite formation by impact mixing of olivine and metal during the first 10 million years of the Solar System

**DOI:** 10.1093/pnasnexus/pgac015

**Published:** 2022-03-09

**Authors:** Richard J Windmill, Ian A Franchi, Jan L Hellmann, Jonas M Schneider, Fridolin Spitzer, Thorsten Kleine, Richard C Greenwood, Mahesh Anand

**Affiliations:** Planetary and Space Sciences, School of Physical Sciences, The Open University, Walton Hall, Milton Keynes, MK7 6AA, UK; Planetary and Space Sciences, School of Physical Sciences, The Open University, Walton Hall, Milton Keynes, MK7 6AA, UK; Institut für Planetologie, University of Münster, Wilhelm-Klemm-Strasse 10, 48149 Münster, Germany; Institut für Planetologie, University of Münster, Wilhelm-Klemm-Strasse 10, 48149 Münster, Germany; Institut für Planetologie, University of Münster, Wilhelm-Klemm-Strasse 10, 48149 Münster, Germany; Institut für Planetologie, University of Münster, Wilhelm-Klemm-Strasse 10, 48149 Münster, Germany; Max Planck Institute for Solar System Research, Justus-von-Liebig-Weg 3, 37077 Göttingen, Germany; Planetary and Space Sciences, School of Physical Sciences, The Open University, Walton Hall, Milton Keynes, MK7 6AA, UK; Planetary and Space Sciences, School of Physical Sciences, The Open University, Walton Hall, Milton Keynes, MK7 6AA, UK; Department of Earth Sciences , The Natural History Museum, London, SW7 5BD, UK

**Keywords:** pallasites, oxygen isotopes, isotope fractionation, Mn–Cr dating, Hf–W dating

## Abstract

Pallasites are mixtures of core and mantle material that may have originated from the core–mantle boundary of a differentiated body. However, recent studies have introduced the possibility that they record an impact mix, in which case an isotopic difference between metal and silicates in pallasites may be expected. We report a statistically significant oxygen isotope disequilibrium between olivine and chromite in main group pallasites that implies the silicate and metal portions of these meteorites stem from distinct isotopic reservoirs. This indicates that these meteorites were formed by impact mixing, during which a planetary core was injected into the mantle of another body. The impactor likely differentiated within ∼1–2 Myr of the start of the Solar System based on Hf–W chronology of pallasite metal, and we infer the age of the impact based on Mn–Cr systematics and cooling rates at between ∼1.5 and 9.5 Myr after Ca–Al-rich inclusions (CAIs). When combined with published slow subsolidus cooling rates for these meteorites and considering that several pallasite groups exist, our results indicate that such impacts may be an important stage in the evolution of planetary bodies.

Significance Statement This study identifies an oxygen isotopic disequilibrium between olivine and chromite in main group pallasites that directly supports an impact formation process for the meteorite group. This addresses a long-standing question in meteoritical science about pallasite origins and provides additional constraints on the timing of differentiation of the impacting body and on the time of the impact itself through Hf–W and Mn–Cr isotope systematics, respectively. These findings imply that impacts between fully differentiated bodies were occurring very early in Solar System history and the presence of several pallasite groups from different nucleosynthetic reservoirs implies that this process may have been Solar System wide.

## Introduction

Despite the rapidly growing catalog of discovered exoplanets and the abundance of rocky planets and moons in our Solar System, the processes active in the earliest stages of planetary formation and evolution remain poorly understood (e.g. 1). The stony-iron and iron meteorites are fragments of disrupted early formed differentiated planetesimals that potentially record deep-mantle and core processes, which cannot be readily observed elsewhere. Iron meteorites reveal that some of these planetesimals underwent core formation contemporaneously with the formation of chondrites and therefore must have accreted earlier, within 1 Myr after the formation of Ca–Al-rich inclusions (CAIs) (2, 3). As such, the samples from these bodies provide key information on both the formation and differentiation processes governing the evolution of planets, as well as on the compositional heterogeneity of the protoplanetary disk and factors such as body sizes and timescales for accretion and differentiation. One type of stony-iron meteorite, the pallasites, are an enigmatic mix of core and mantle materials. They, therefore, offer a unique window into the processes that were active deep in planetesimals during the earliest stages of formation and evolution of protoplanetary bodies and have the potential to provide information about core–mantle mixing processes that we cannot directly observe on Earth. The most numerous pallasite group is the main group pallasites, accounting for ∼80% of approved pallasites that have been assigned to a chemical group (4).

Main group pallasites are slowly cooled (5) mixtures of olivine and Fe-Ni metal in approximately equal quantities by mass (6, 7), although some pallasites can exhibit considerable variation in olivine–metal ratio. Many pallasite formation models involve the partial melting and/or crystallization of chondritic material (8–11) situated at the core–mantle boundary (12), or the collapse of mantle material into the core of a planetesimal (13). Until relatively recently, a core–mantle boundary origin was the most popular model for the formation of the main group pallasites (e.g. 14, 15), although other mechanisms such as mixing by collapse of dunite into metal pods (16), double impacts (17), and ferrovolcanism (18, 19) have also been proposed (see 20 for partial review). Recently, the idea that pallasites are the products of impact mixing has gained traction (21, 22). The discovery of large variations in the cooling rate of the metal (5) and the identification of palaeomagnetic signatures in pallasite olivine that suggest formation in the upper 60% of a planetesimal (21) support an impact mechanism for pallasite formation. The question of whether the silicate and metal portions of main group pallasites are derived from one or more planetary bodies is a key outstanding question in meteoritical science and has significant implications for our understanding of the early formation and evolution of rocky planetesimals. The models that invoke an impact mixing event can be directly tested using high-precision oxygen isotope analyses.

As a result of its abundance and its relatively high oxygen content, olivine has been the mineral of choice for oxygen isotope analyses on pallasites in numerous studies (23–26). Olivine is not, however, the only oxygen-bearing phase found these meteorites. Chromite is found in low quantities in many main group pallasites but has not been analyzed for oxygen isotopes. The origin of the metal and silicate phases in pallasites and the relationship between these 2 components remains unclear. However, the presence of near endmember chromites [FeCr_2_O_4_] in some samples that may have crystallized from the metal raises the possibility for a direct isotopic comparison of silicate and metal phases. This study presents a detailed investigation into olivine and chromite oxygen isotopic compositions to test the veracity of recent impact models for pallasite formation. In addition, these data are put in chronological context by Mn–Cr and Hf–W systematics.

Bulk meteorites exhibit mass-independent oxygen isotope variations so that each parent body has a distinct Δ^17^O (see Materials and Methods for definition) and therefore oxygen isotope analyses have tremendous utility as a genetic tracer (e.g. 23, 24, 27–29). The effects of melting and planetary differentiation lead to homogenization of the isotopic signatures within planetary bodies. Subsequent processes (e.g. crystallization) affecting the isotopic signatures are usually small and are well-constrained to mass-dependent signatures that can readily be resolved from the mass-independent heterogeneity between planetary bodies by high-precision oxygen 3 isotope measurements. The olivines in pallasites conform to this scenario, displaying isotopic variation only consistent with mass-dependent fractionation after complete homogenization (24, 25), although it has recently been suggested that some isotopic heterogeneity may persist (26).

Chromium isotopes have also been used to investigate differences in meteorite provenance, particularly in conjunction with O and Ti isotopes (e.g. 30). To this end, bulk meteorites are characterized by variable ε^54^Cr values (see Materials and Methods for definition). In a plot of ε^54^Cr vs. Δ^17^O, meteorites can be subdivided into 2 clusters, termed the non-carbonaceous (NC) and carbonaceous (CC) reservoirs. Meteorites from each cluster display broadly correlated variations of ε^54^Cr and Δ^17^O, making combined Cr–O isotope analyses a powerful tool to identify genetic differences among meteorites and their components. Furthermore, Cr isotope measurements allow application of the short-lived ^53^Mn–^53^Cr decay system (*t*_1/2_ = 3.7 ± 0.4 Myr; 31) to pallasites, which combined with the high Mn/Cr ratio of pallasite olivine makes it possible to determine precise cooling ages. By contrast, the short-lived ^182^Hf–^182^W decay system (*t*_1/2_ = 8.9 ± 0.1 Myr; 32) provides constraints on the timescale of metal–silicate fractionation (33, 34) and, hence, the formation time of pallasite metal.

Here we report O, Cr, and W isotopic compositions of olivine and chromite in main group pallasites and directly test recent core injection models as well as date key events in their formation.

## Results

Olivines from all pallasites analyzed in this study were categorized based on texture either from available samples or published literature. In addition, olivine and chromite phases in slices of Seymchan, Sericho, and Fukang as well as a piece of chromite from Brenham were analyzed in detail using EPMA to provide context for the isotopic analyses.

### Olivine petrology—texture and composition

Pallasite olivines exhibit textural differences that can be present both between different meteorites but also within individual meteorites between one grain and the next (14, 15, 20, 35). Textural classification of pallasite olivines identifies 3 groups: fragmental, rounded, and angular (7, 14, 15, 20). There is generally good agreement between the textural classification of the samples analyzed in this study and published classifications (e.g. 14, 35). The exception to this is Fukang, which has previously been classified as angular with rounded grains (35) whilst only angular olivine was visible in the slice studied here. The pallasite olivines analyzed for major element compositions in this study are restricted to Sericho, Seymchan, and Fukang (Table S1, Supplementary Material). Analyses were performed on olivine cores, olivine–chromite rims, and olivine–metal rims.

### Chromite petrology—texture and composition

Our data and literature data show that chromites in main group pallasites have very diverse Al contents (Tables S2 and S3, Supplementary Material), which correlate with several other properties (20, 35, 36). There appear to be 2 subgroups of main group pallasites: one in which the chromites have very low Al contents (0–2 wt% Al_2_O_3_) and correspondingly high Cr contents, and a second with variable to high Al chromite (3–20 wt% Al_2_O_3_) and correspondingly lower Cr contents (Table S2, Supplementary Material). This diversity is likely controlled by the amount of silicate melt present during crystallization (20), low Al chromite having likely crystallized from the metal in the absence of appreciable silicate melt, whereas high Al chromites crystallized from silicate melt and not metal. In the Al-rich chromites, Cr_2_O_3_ content is typically higher toward rims than in the cores, with a corresponding opposite trend observed for Al_2_O_3_. This is most pronounced in Fukang (Figure S1, Supplementary Material).

**Fig. 1.**
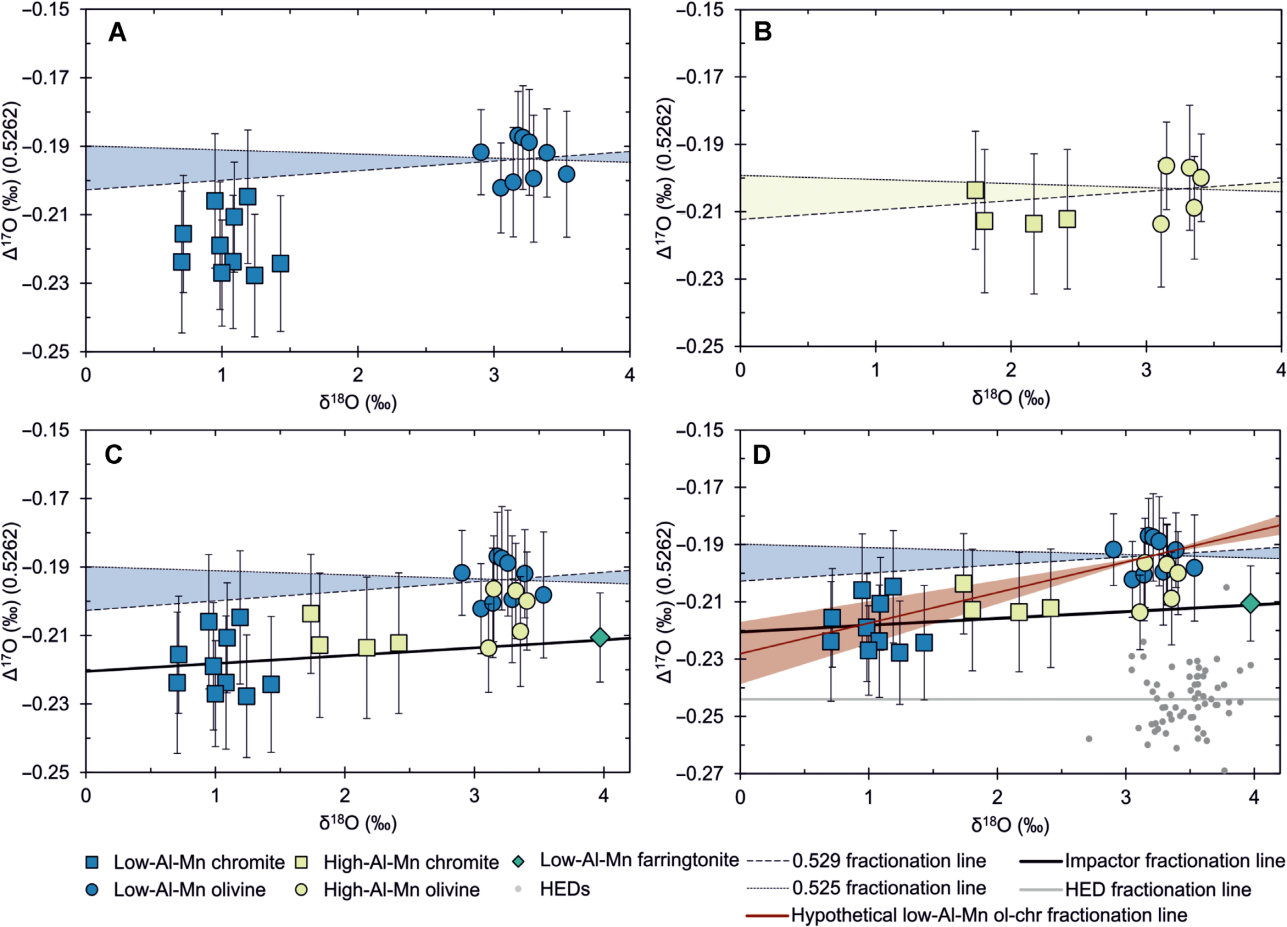
Oxygen isotope results for main group pallasite mineral analyses. (A) Low-Al–Mn chromite and olivine results. The blue shaded region is the envelope of typical mass-dependent fractionation (0.525–0.529) accessible from the olivine. The chromite would, therefore, be expected to plot in this shaded area if in equilibrium with the olivine. The offset that is present is inexplicable through any mass-dependent means. (B) High-Al–Mn chromite and olivine results. The yellow shaded area is the envelope of typical mass-dependent fractionation from high-Al–Mn subgroup olivine (0.525–0.529). Note that the high-Al–Mn chromite in panel B are within error of the envelope, whereas the low-Al–Mn chromite in panel A are generally well outside of the envelope. High-Al–Mn chromite seems to record mixing or partial equilibration between the olivine and low-Al–Mn chromite reservoirs. (C) Olivine and chromite from both subgroups plotted together. The blue shaded area is again the envelope of mass-dependent fractionation (0.525–0.529) relative to low-Al–Mn subgroup olivine. Farringtonite from Sericho (low-Al–Mn subgroup) is shown as a green diamond. The solid black line is a trendline through the low-Al–Mn subgroup chromite and farringtonite data and may represent the mass-fractionation line of the impactor metal. The olivine mantle material from the target body is offset from this line. (D) All data from panel C but with measurements from HED meteorites added to illustrate how crowded this area of isotope space is. HED data are taken from (39–42). The red line is slope required to connect the low-Al–Mn olivine and chromite and in linearized δ^17^O-δ^18^O space is 0.5369 ± 0.0034 (2 SEM). The 2 SE range on this slope is shown as a red shaded area. The individual HED samples are shown as grey circles, the HED fractionation line is shown as a solid grey line. The data shown here are from five pallasites with low-Al chromite and two pallasites with high-Al chromite (see Tables 1 and 2). Y-axis errors are 2 SEM; X-axis errors (2 SEM) are smaller than the symbols.

Chromite texture can vary from large polycrystalline masses (e.g. 36) to small, relatively angular chromite grains in pallasites. Texture is not a reliable indicator of chromite petrogenesis, however. This is apparent because chromite in core-derived magmatic irons can range from globular (e.g. Fig. 1477 in (37)) to very angular crystals (e.g. Fig. 1 in (38)). As a result of this, chromite textures are not considered when interpreting main group pallasite petrogenesis in this study.

### Oxygen isotope results

A total of 14 samples of olivine and 14 samples of chromite were analyzed from 7 different pallasites as well as a single grain of farringtonite [Mg_3_(PO_4_)_2_] from Sericho. The results are displayed in Table 1. In addition, olivine results from a larger sample group are presented and discussed in the SI and compared to the olivine results from previously published work.

**Table 1.** Oxygen isotope results for analyzed pallasite olivines.

Pallasite	O (wt%)	δ^17^O	2 SE	δ^18^O	2 SE	Δ^17^O	2 SE	n
Brahin	45.0	1.531	0.018	3.292	0.005	−0.199	0.019	60
Brenham	42.5	1.483	0.013	3.177	0.004	−0.187	0.013	60
Brenham	43.9	1.660	0.018	3.535	0.005	−0.198	0.018	60
*Fukang*	45.1	1.421	0.013	3.110	0.006	−0.214	0.013	60
*Fukang*	41.8	1.459	0.015	3.149	0.004	−0.196	0.015	60
*Fukang*	40.5	1.590	0.014	3.406	0.005	−0.200	0.014	60
Hambleton	44.4	1.501	0.015	3.213	0.005	−0.187	0.015	60
Hambleton	45.2	1.590	0.012	3.389	0.005	−0.192	0.013	60
*Imilac*	39.6	1.548	0.014	3.320	0.008	−0.197	0.014	60
*Imilac*	44.1	1.555	0.016	3.356	0.004	−0.209	0.016	60
Sericho	46.0	1.402	0.012	3.051	0.006	−0.202	0.013	60
Sericho	25.1	1.451	0.016	3.142	0.010	−0.200	0.016	60
Seymchan	39.5	1.335	0.012	2.904	0.005	−0.192	0.012	60
Seymchan	44.3	1.524	0.016	3.259	0.005	−0.189	0.015	60
Avg(all)	41.9	1.504	0.172	3.236	0.327	−0.197	0.016	
Avg(low-Al–Mn)		1.498	0.193	3.218	0.368	−0.194	0.011	
Avg(high-Al–Mn)		1.515	0.071	3.268	0.262	−0.203	0.015	

High-Al–Mn subgroup samples are in italics. The errors on the average populations (underlined) are 2 SD. O (wt%) refers to the amount of oxygen generated during laser fluorination as a percentage of the sample weight. The number of analyses of each sample gas aliquot vs. the reference gas is shown in the “n” column.

The olivine data show a homogenous Δ^17^O composition consistent with the findings of previous studies (e.g. 24, 25); Δ^17^O = −0.197 ± 0.016 ‰ (2σ; Table 1), and at comparable precision. The errors (2σ) reported on pallasite olivine Δ^17^O values by Greenwood et al. (24, 25) are in line with this study at ± 0.018 ‰ and ± 0.016 ‰, respectively. Neither this study, nor the two by Greenwood et al. (24, 25) have found evidence for the oxygen isotope bimodality in the olivines as reported by Ali et al. (26) (Figures S4 and S5, Supplementary Material); the difference between the high Δ^17^O and low Δ^17^O subgroups reported by (26) is 0.054 ‰, which approaches 7 SDs at the precision of the olivine population in this study. The reason for this discrepancy is explored in the supplementary information but is currently unknown. The δ^18^O variations for olivines (± 0.33 ‰; Table 1) are higher than analytical error (δ^18^O = ± 0.16 ‰, see Materials and Methods) indicating there is some small variability across the population but as the errors on Δ^17^O results (± 0.016‰) are smaller than the normal analytical precision (± 0.021 ‰), the olivine is homogenous with respect to Δ^17^O and variations in δ^18^O are all mass dependent.

The average composition of Al_2_O_3_ enriched chromites is δ^18^O = 2.03 ± 0.64 ‰ (2σ), and Δ^17^O = −0.211 ± 0.009 ‰ (2σ). The average composition of chromites with low Al_2_O_3_ contents is δ^18^O = 1.04 ± 0.45 ‰ (2σ), and Δ^17^O = −0.218 ± 0.017 ‰ (2σ; see Table 2). Crucially, the Δ^17^O results for chromites containing low Al_2_O_3_ are offset from the olivines in the same samples (Fig. 1). Both chromite subgroups show mass-dependent internal variability in both δ^17^O and δ^18^O, but are homogenous in Δ^17^O and appear resolvable from each other in both δ^18^O and Al_2_O_3_ vs. Cr_2_O_3_ abundances (Fig. 1 and Figure S2, Supplementary Material). The high Al_2_O_3_ chromite is part way between the low Al_2_O_3_ chromite and the olivine composition (Fig. 1). The farringtonite (Fig. 1 and Table 2) from Sericho also appears to be offset toward lower Δ^17^O values relative to the olivine, δ^18^O = 3.971 ± 0.004 (2 SE), and Δ^17^O = −0.211 ± 0.013 (2 SE) but was a single sample and is therefore relatively unconstrained.

**Table 2.** Oxygen isotope results for farringtonite and chromite.

Pallasite	O (wt%)	δ^17^O	2 SE	δ^18^O	2 SE	Δ^17^O	2 SE	n
Sericho*	45.3	1.876	0.013	3.971	0.004	−0.211	0.013	60
Brahin	26.7	0.293	0.019	0.949	0.008	−0.206	0.020	60
Brenham	25.5	0.421	0.018	1.190	0.008	−0.205	0.019	60
Brenham	26.8	0.161	0.017	0.716	0.007	−0.216	0.017	60
Brenham	28.0	0.147	0.020	0.705	0.007	−0.224	0.021	60
*Fukang*	20.3	0.737	0.020	1.805	0.010	−0.213	0.021	60
*Fukang*	28.1	1.058	0.020	2.416	0.008	−0.212	0.021	60
Hambleton	27.4	0.425	0.018	1.241	0.008	−0.228	0.018	60
Hambleton	26.2	0.299	0.019	0.985	0.007	−0.219	0.019	60
*Imilac*	28.5	0.710	0.017	1.737	0.005	−0.204	0.017	60
*Imilac*	29.5	0.927	0.019	2.169	0.007	−0.214	0.021	60
Sericho	28.4	0.528	0.020	1.431	0.007	−0.224	0.020	60
Sericho	22.4	0.345	0.020	1.081	0.008	−0.224	0.019	60
Seymchan	27.8	0.299	0.015	1.000	0.006	−0.227	0.015	60
Seymchan	28.9	0.363	0.017	1.090	0.008	−0.211	0.016	60
Avg(low-Al–Mn)		0.328	0.234	1.039	0.447	−0.218	0.017	
Avg(high-Al–Mn)		0.858	0.234	2.032	0.637	−0.211	0.009	

High-Al–Mn subgroup samples are in italics. The Sericho farringtonite is marked with an asterisk. The errors on the average populations (underlined) are 2 SD. O (wt%) refers to the amount of oxygen generated during laser fluorination as a percentage of the sample weight. The number of analyses of each sample gas aliquot vs. the reference gas is shown in the “n” column.

### Chromium isotopes

**Fig. 2.**
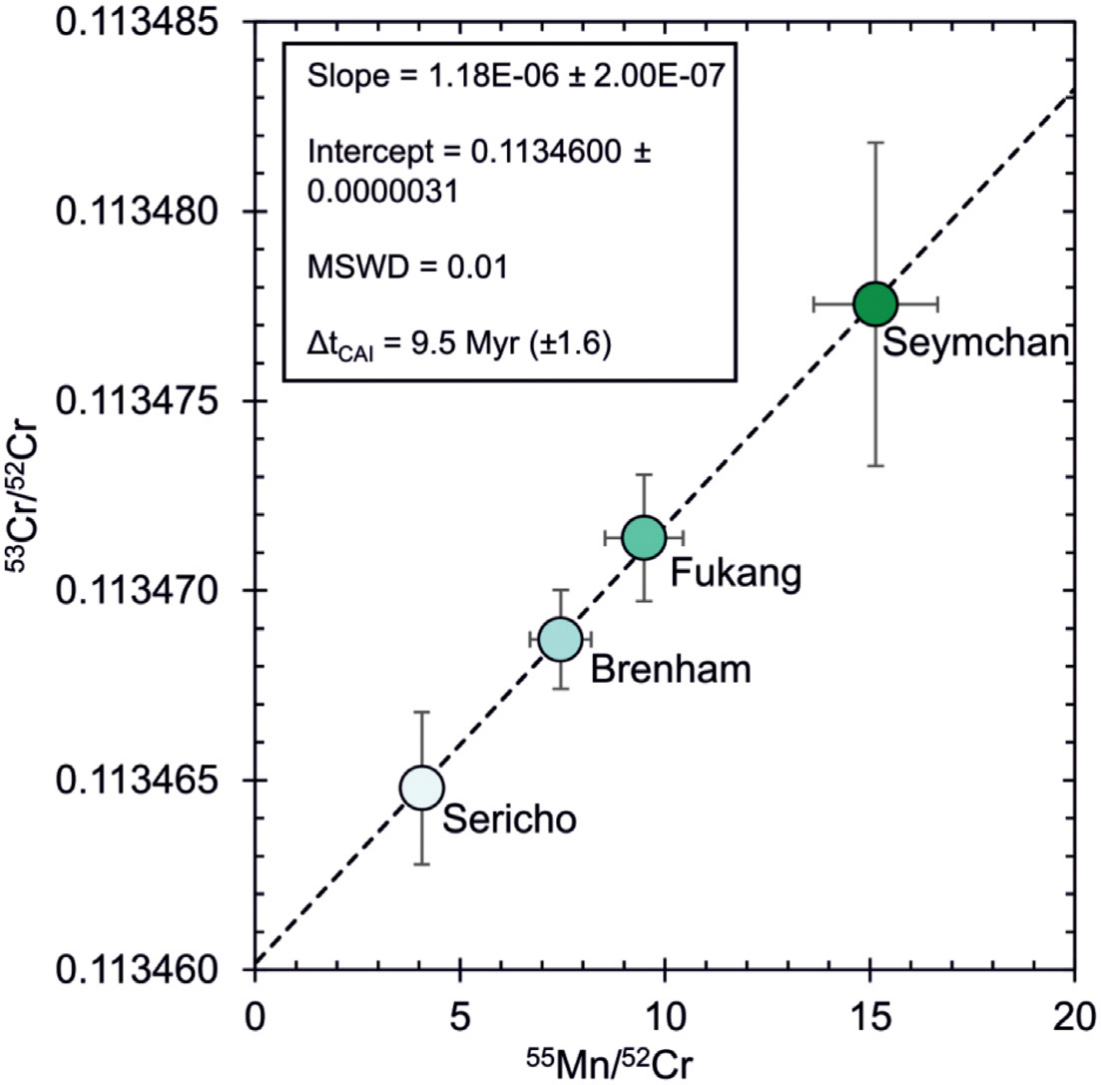
An isochron for spallation-corrected olivines. Isochron redrawn from one generated using ISOPLOT following GCR correction to the average of enstatite chondrite ε^54^Cr (47) and ureilites (48). *X*-axis errors are 10%, *Y*-axis errors are 2 SEM. Brenham data from (49).

The Cr isotope compositions of 4 samples of olivine and 5 samples of chromite were analyzed from 5 different pallasites (Table 3). The ε^54^Cr and ε^53^Cr values of the chromites are homogeneous, and only the ε^54^Cr of Hambleton chromite seems to be slightly offset but still within error of other pallasites. By contrast, the ε^54^Cr and ε^53^Cr of the olivines are more variable and are distinct from the values of chromite from the same samples. These Cr isotope variations can have different origins and may record compositional differences in precursor materials (30), but can also be affected by processes such as cosmic ray spallation and ^53^Mn–^53^Cr decay. Spallation is significant for samples with high Fe/Cr ratios, such as olivines, predominantly affects ε^54^Cr, and results in correlated ε^54^Cr–ε^53^Cr variations with a characteristic slope of ∼3.9 (43). By contrast, chromite has low Fe/Cr and spallation effects on Cr isotopes are, therefore, virtually absent. Hence, the distinct ε^54^Cr values of olivine and chromite from a single pallasite partly or wholly reflect cosmic ray induced spallation on Cr isotopes in olivine. As a result, ε^54^Cr cannot be used to identify a potential nucleosynthetic isotope difference between olivine and chromite. The ε^54^Cr data can, however, be used for spallation correction of measured ε^53^Cr values for pallasite olivine (see Supplementary Information). After spallation correction, the ε^53^Cr value of pallasite olivine is directly linked to the Mn/Cr ratio of the sample, indicating that these ε^53^Cr variations are radiogenic in origin, i.e. that they reflect early Mn–Cr fractionation and subsequent ^53^Mn-decay. The spallation-corrected ε^53^Cr data, therefore, allow the determination of the time of Mn–Cr closure in the olivine samples (Fig. 2). The Mn–Cr isochron in Fig. 2 was generated using the ISOPLOT add-in for Microsoft Excel and using the initial ^53^Mn/^55^Mn and absolute Pb–Pb age of D'Orbigny as a time anchor (44, 45) to convert the relative Mn–Cr ages to absolute ages. Ages relative to the formation time of CAIs are given assuming a Solar System initial ^53^Mn/^55^Mn ratio of (7 ± 1) x10^–6^ from (46), which is based on a compilation of available Mn–Cr, Hf–W, and Pb–Pb age data for angrites and CAIs.

**Table 3.** Cr isotope results for analyzed olivine (Ol) and chromite (Chr).

Meteorite	Phase	Fe/Cr	Mn (ppm)	Cr (ppm)	^55^Mn/ ^52^Cr	± (10%)	ε^53^Cr	±	ε^54^Cr	±	ε^53^Cr _CORR_	±	53/52Cr (±)
Seymchan	Ol	695.8	2,641	197	15.14	1.51	1.69	0.36	0.14	0.50	1.55	0.38	0.1134775 (0.0000043)
Sericho	Ol	270.9	1,259	353	4.07	0.41	0.54	0.14	0.04	0.08	0.42	0.18	0.1134648 (0.0000020)
Fukang	Ol	349.1	2,949	353	9.49	0.95	1.07	0.10	−0.16	0.19	1.00	0.15	0.1134714 (0.0000017)
*Brenham*	Ol				7.46	0.75	0.86	0.04	−0.06	0.11	0.77	0.11	0.1134687 (0.0000013)
**Average**	**Ol**						**1.04**	**0.97**	−**0.01**	**0.26**			
Hambleton	Chr						−0.15	0.07	−0.8	0.10			
Seymchan	Chr	0.31	4,800	484,500	0.011	0.001	−0.09	0.07	−0.53	0.13			0.1134590 (0.0000008)
Sericho	Chr	0.33	3,900	489,800	0.009	0.001	0.03	0.07	−0.40	0.13			0.1134603 (0.0000008)
Fukang	Chr	0.45	3,900	339,700	0.013	0.001	0.04	0.10	−0.57	0.20			0.1134605 (0.0000011)
Brenham	Chr	0.30	3,400	492,400	0.008	0.001	−0.04	0.07	−0.44	0.13			0.1134595 (0.0000008)
**Average**	**Chr**						−**0.04**	**0.16**	−**0.49**	**0.31**			

The spallation corrected ε^53^Cr values are the mean of the corrected values calculated using the ureilites and enstatite chondrites as initial ε^54^Cr compositions for olivines. For the chromites, the spallation correction was not required due to the very low target nucleus/Cr ratio. Brenham olivine values are taken from (42). The errors on the average values (underlined) are 2 SD, errors on samples are 2 SE.

The Mn–Cr isochron comprising just the spallation-corrected olivines (Fig. 2) differs from previously reported isochrons in that they incorporate both chromite and olivine (e.g. 50). Figure 2 yields an initial ^53^Mn/^55^Mn of (1.18 ± 0.20) × 10^–6^ (2σ) with an MSWD of 0.01. Including the chromites in the isochron regression has little effect on the slope [(1.21 ± 0.17) × 10^–6^] and shows no excess scatter (MSWD = 0.32). Thus, the olivines and chromites plot on a single, well-defined isochron, which yields an age of 4558.1 ± 1.0 Ma (2σ) or Δt_CAI_ = 9.4 ± 1.5 (2σ) Myr, consistent with the isochron age reported for olivine and chromite from the Omolon pallasite by (50). Note that the oxygen isotope results of this study indicate that there is no simple chemical relationship and isotopic equilibrium between the low Al_2_O_3_ chromites and the co-occurring olivines. As such, these samples may not define a single isochron. However, the overall much larger O than Cr isotope variations among meteorites and their components, combined with the very small O isotope offset between olivines and chromites reported in this study, implies that any pre-existing ^53^Cr heterogeneity between chromites and olivines is negligible and that these minerals had essentially the same initial ε^53^Cr. This is consistent with the observation that excluding the chromites from the isochron regression does not significantly change the slope of the isochron or the age [Δt_CAI_ = 9.5 ± 1.6 Myr (2σ)].

### Tungsten and Pt isotope compositions of main group pallasite metals

Tungsten and Pt isotope compositions of metal from the pallasites Fukang and Seymchan are reported in Table S4 (Supplementary Material). The cosmic-ray exposure (CRE) corrected W isotope data and Hf–W model ages are presented in Table 4. The pre-exposure ε^182^W values have been calculated following the approach described in (3) (see Supplementary Information for details). The model age of metal segregation can then be calculated as the time of Hf–W fractionation from an unfractionated reservoir with chondritic Hf/W using the following equation (e.g. 51):

(1) $$\begin{equation*}
\Delta \ {t_{CAI}} = {\rm{\ }} - \frac{1}{\lambda } \times ln\left[ {\frac{{{\varepsilon ^{182}}{W_{sample}} - {\varepsilon ^{182}}{W_{chondrites}}}}{{{\varepsilon ^{182}}{W_{SSI}} - {\varepsilon ^{182}},{W_{chondrites}}}}} \right],
\end{equation*}
$$

where ${\varepsilon ^{182}}{W_{sample}}$ is the W isotope composition of pallasite metal, ${\varepsilon ^{182}}{W_{chondrites}}$ is the composition of CC chondrites (−1.9 ± 0.1) (34), ${\varepsilon ^{182}}{W_{SSI}}\ $ is the Solar System initial composition (−3.49 ± 0.07) obtained from CAIs (2), and $\lambda $ is the decay constant of ^182^Hf of 0.0778 ± 0.0015 Myr^–1^(32). The pre-exposure ε^182^W value for Seymchan (−3.31) corresponds to a Hf–W model age of (see SI for calculation of pre-exposure ε^182^W) 2.2 ± 1.2 (2σ) Myr after CAI formation, whereas this age is 1.1 ± 1.1 Myr (2σ) for Fukang (Table 4). These ages are unresolvable from each other.

**Table 4.** Pt corrected W isotope results for Seymchan and Fukang with differentiation ages.

Sample	ε^182^W (6/4)	95% conf	ε^183^W (6/4)	95% conf	ΔT_CAI_ [Myr]	2σ
Seymchan	−3.24	0.11	0.04	0.07	2.2	1.2
Fukang	−3.36	0.11	0.23	0.10	1.1	1.1

## Discussion

### Subgroups

To provide context for the interpretation and conclusions presented in this study, the pallasites have been assigned subgroups based on texture and major element composition of olivine and chromite. For simplicity, 2 subgroups are defined combining features identified by (35) with isotopic data obtained during this study and named for the relative Al content in the chromite and Mn content in the olivine. Where used to differentiate minerals belonging to these subgroups, the names refer to which subgroup the minerals belong and are not describing the composition of the mineral. For example, low-Al–Mn olivine indicates that the olivine is from a pallasite wherein the chromite has low-Al_2_O_3_ and the olivine has low-MnO.

A crucial finding is that the low-Al–Mn chromites are offset in Δ^17^O relative to olivines from the same samples in a manner that is not possible through any known fractionation process (Fig. 1). A regression through the linearized δ^17^O and δ^18^O values (see Materials and Methods for definition) for these minerals yields a slope ($\lambda $) of 0.5369 ± 0.0017. This is ∼4 SE above the maximum possible mass-fractionation slope for oxygen isotopes, 0.5305, and ∼5 SE above typical igneous $\lambda $ values of 0.525–0.529 (see (52); see Fig. 1).

### Statistical testing

A student's t test was conducted on the calculated Δ^17^O for olivine and chromite of each subgroup using a $\lambda $ input of 0.5262 (Table S5, Supplementary Material). The Δ^17^O difference between low-Al–Mn chromite and corresponding olivine is statistically significant with a *P*-value < 0.0001. For the high-Al–Mn subgroup, the *P*-value is not statistically significant at 95% CI (Table S5, Supplementary Material), consistent with the chromite from this subgroup representing some degree of equilibration between the silicate melt from which the olivine crystallized and the low-Al–Mn chromite isotopic reservoir.

In geological systems, the $\lambda $ value for oxygen is not unique and varies depending on the temperature and fractionation process (e.g. 52). In order to account for this potential variability, and to ensure that the use of an inappropriate fractionation exponent was not responsible for the observed disequilibrium, a student's t test on these data and a student's t test with the *P*-value computed using a Monte Carlo simulation on 10^6^ samples (Table S5, Supplementary Material) were also carried out in XLSTAT using the built in function. This was done on the Δ^17^O differences between olivine and chromite in both subgroups calculated using a fractionation exponent of 0.5305, the high-T upper limit (52–54). The Δ^17^O offset between the olivine and chromite minerals in low-Al–Mn pallasites is highly statistically significant (*P* = 0.001) even when this fractionation exponent is used and remains so for the simulated 10^6^ datapoints. This demonstrates that the offset between the olivine and chromite in this subgroup is highly statistically significant at all fractionation exponents possible through mass-dependent processes, and therefore, an oxygen isotope disequilibrium is present.

### Origin of the oxygen isotope disequilibrium

We interpret the observed oxygen isotope disequilibrium as recording the mixing of 2 different isotopic reservoirs during a planetary impact, however, other factors such as anharmonic effects, the nuclear field shift effect, and cosmic ray spallation could also possibly give rise to the observed offset. The possibility of such processes affecting the results is discussed in the Supplementary Information, but each has been discounted or thought to be very unlikely.

### Multiple parent bodies

The discovery in this study of a statistically significant isotopic Δ^17^O disequilibrium between low-Al–Mn chromite and associated olivine is not explicable through any known mass-dependent process. This finding is inconsistent with single-body models for pallasite genesis such as ferrovolcanism (e.g. 18) or fractional melting (e.g. 20) at a core–mantle boundary (e.g. 14, 15), where mass dependent fractionation effects will dominate. The low-Al–Mn chromite, which crystallized from the intruded metal, and the olivine into which the metal was injected sample distinct oxygen isotopic reservoirs and therefore appear to be recording the mixing of 2 planetary bodies. This directly supports previously proposed impact injection models for metal–olivine mixing (21). The fractionation exponent, or slope of mass-fractionation between phases on a 3-oxygen isotope diagram, can vary between 0.5000 and 0.5305 (52–54). In practice, the range over which this value varies is more restricted, ∼0.525 to ∼0.529 for high temperature igneous processes (52). Indeed, empirically determined fractionation exponents for terrestrial igneous materials have yielded values of 0.5281 ± 0.0025 and 0.529 ± 0.006 for granitic and MORB/Earth mantle minerals, respectively (55). As mentioned previously, for the olivine and chromite analyzed from the low-Al–Mn subgroup, the slope in 3 oxygen isotope space is 0.5369 ± 0.0017 (SE); ∼5 SE above previously reported values for terrestrial igneous minerals (55), and the relationship between these minerals is, therefore, inexplicable in mass dependent terms. The high-Al–Mn chromite and associated olivine on the other hand, do not exhibit the same statistically significant disequilibrium and the slope ($\lambda $) is within error of expected bounds for high-T mass-fractionation. Coupled with the higher Al content and the marked decrease in Al_2_O_3_ and increase in Cr_2_O_3_ from core to rim in Fukang chromite (Figure S1, Supplementary Material), this is interpreted as evidence that high-Al–Mn chromite formed during partial equilibration between the isotopic reservoirs represented by the olivine and low-Al–Mn chromite. Based on their high-Al_2_O_3_ cores, high-Al–Mn chromites may have initially crystallized as a mantle cumulate mineral with the pallasite olivine. Injection of the metal could then have displaced an Al-bearing silicate melt or intercumulus liquid and resulted in the continued growth of chromites incorporating their major and minor element chemistry from the metal. This might explain the strong increase in Cr/Al from core to rim in Fukang chromite and the oxygen isotope data that seems to be intermediate between low-Al–Mn chromite and olivine values. The fact that the low-Al–Mn chromites are homogenous with respect to Δ^17^O implies that either no oxygen was scavenged from the olivine during low-Al–Mn chromite crystallization or that the conditions under which oxygen was scavenged were identical across all low-Al–Mn samples. Given that, for example, Sericho and Seymchan exhibit variable olivine–metal ratios yet have unresolvable Δ^17^O values, it does not seem likely that low-Al–Mn chromite scavenged a significant amount of oxygen from olivine during crystallization.

**Fig. 3.**
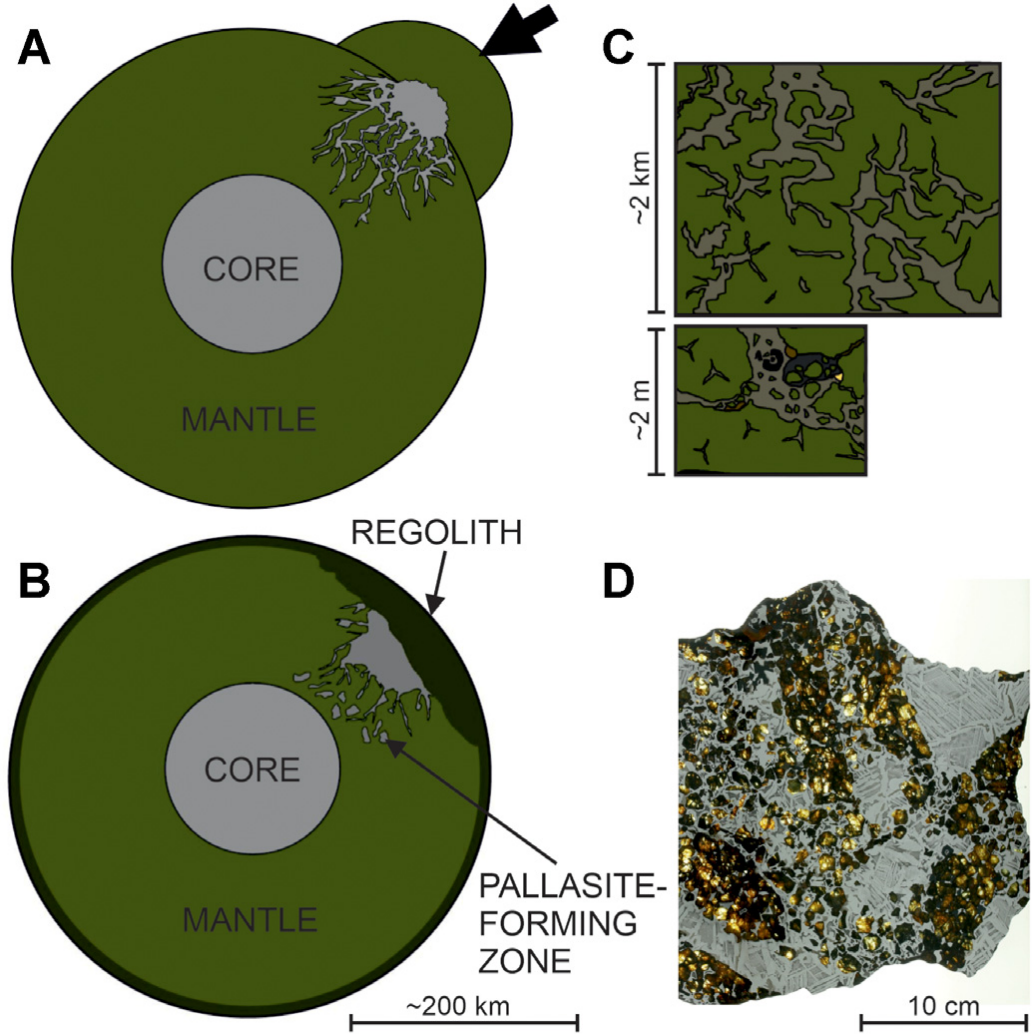
Impact injection model for pallasite formation similar to one proposed previously by (21). This figure is separated into 4 parts: (A) impact between 2 differentiated bodies, the core of the smaller body is injected into the mantle of the larger body. (B) Over time, an insulating layer of regolith develops and the silicate and liquid metal cool together at depth. (C) At the km scale, the metal–silicate distribution is heterogeneous, with some large metallic veins and some large olivine clusters. At the meter scale, pallasite textures are as observed in meteorites. This depicts chromite (black mineral) and troilite (gold mineral) crystallizing from the metal during cooling. (D) An image of a pallasite at submeter scale displaying the same textures seen in C, image credit: Luc Labenne.

Integrating the O isotope results with the Mn–Cr and Hf–W chronologies allows a detailed formation model to be developed (Fig.   3), which describes the impact injection of the core of a differentiated planetesimal (the impactor) into the mantle of another, larger, differentiated body (the target). In this model, olivine from both subgroups is isotopically representative of the mantle of the impacted body. The low-Al–Mn chromite is an isotopic record of the composition of the impactor core, incorporating O dissolved in the metal. The high-Al–Mn chromite formed first in the mantle of the impacted body, then subsequently grew in the impactor metal following displacement of an Al-bearing silicate melt. This model suggests that pallasites incorporate material from 2 distinct parent bodies and is consistent with an array of observed characteristics. The initial impact injection stage of this process (Fig. 3A) is probably followed by burial of the impact crater with regolith (Fig. 3B). The impact must have been between 2 fully or near fully differentiated bodies to account for the relative internal Δ^17^O homogeneity of the 2 reservoirs reflected by the olivine and low-Al–Mn chromite. The impact energy must also have been very high to allow for the injection of core material to tens of km depth as required by slow metallographic cooling rates (5). At the km scale, metal dykes crosscut the parent body mantle (Fig. 3C). Such heterogeneity in metal–silicate ratio has been observed across pallasite meteorites; an example of this is Seymchan, which was initially classified as a IIE iron until reclassification in 2007 (4). This raises the intriguing possibility that some iron meteorite groups may be sampling large metal structures such as depicted in Fig. 3. The formation of pallasite texture on the meter scale is also shown in Fig. 3(C). Here, the mantle olivine is shown in association with the intruded Fe-Ni metal as well as chromite and troilite that crystallized from the intruded melt which therefore capture the isotopic composition of a different oxygen reservoir.

The progressive influx of metal is likely what gives rise to the formation of rounded olivines (35, 56) by invading olivine masses and breaking them apart. This process isolates the grains in the metal and allows them to become rounded. The identified relationship between amount of fragmental olivines and metal melt evolution (20) is consistent with an impact injection model. If fragmental olivine is formed by the breakup of pre-existing angular and rounded olivine by impact shockwaves (20), then it would be expected that the proportion of olivine displaying fragmentation should increase closer to the surface. In this model, the metal closer to the surface should have cooled more quickly and therefore is likely to be more primitive than metal at depth, which cooled more slowly. This relationship is exactly what has been reported ( 20). Finally, the fact that the parent body had a continued core dynamo and therefore a convecting core up until the metal cooled below ∼360°C ( 21) is better explained in an impact injection model than in either a core–mantle boundary setting or other hit-and-run style impact models (e.g. 5). In impact–injection models, the pallasites are cooling in a well-insulated environment at a relatively large distance from the planetesimal's convecting core. This is required for the core to be hot enough to allow convection to generate a dynamo and for the pallasites to simultaneously be ∼360°C. Such a temperature differential is difficult to envisage in a core–mantle boundary setting adjacent to the core, although this might be possible in large bodies. Likewise, the complete destruction and reaggregation of a pallasite body proposed by ( 5) seems to be incompatible with a continued core dynamo until ∼360°C . The Sericho (low-Al–Mn) farringtonite results also appear to be offset from olivine in Δ^17^O (Fig. 1), although only 1 sample was analyzed so this must be treated cautiously. Assuming that the farringtonite crystallized from the metal (e.g. 57), and therefore, like the chromite, is isotopically representative of the impactor core, the core-formed minerals sit on a fractionation line of slope 0.5285 (Fig.   1C), a value typical for high temperature igneous processes (e.g. 52, 55), and the olivine mantle of the impacted body shows a positive offset in Δ^17^O. The very similar oxygen isotopic compositions of the olivine and metal-hosted minerals suggests that the 2 planetesimals involved in the collision likely had proximal feeding zones. Given that the nucleosynthetic isotope compositions of main group pallasites are consistent with the NC reservoir ( 3, 30), it seems highly likely that these feeding zones were sunward of Jupiter.

### Chronology

#### Accretion and differentiation

Accretion and differentiation of planetary bodies appears to have occurred very early in the history of the Solar System (2, 3, 34). The Hf–W model ages for Seymchan and Fukang obtained in this study further support this assertion and show that differentiation of the impacting body from which the metal is derived took place between 1.1 ± 1.1 Myr (2σ) and 2.2 ± 1.2 Myr (2σ) after the formation of CAIs. In the context of the impact injection model, these ages refer to the differentiation of the impactor core and not that of the target body from which the olivine is derived.

#### Dating the impact

The impact mixing of pallasites must have occurred after the differentiation of the planetesimals involved. This is because the olivines, representing the impacted body's mantle, are isotopically homogeneous with respect to Δ^17^O, suggesting that melting and homogenization had already occurred. Similarly, the homogeneity in Δ^17^O of the impactor core recorded by the low-Al–Mn chromites is indicative of a relatively well-homogenized oxygen isotopic reservoir. The impact event can be broadly constrained using an exponential cooling rate model (detailed in the Supplementary Information), the date obtained from the closure of the ^53^Mn–^53^Cr decay system, and some assumptions about initial conditions. The Mn–Cr system in the olivines closed 9.5 (± 1.6) Myr after CAI formation. This age likely records cooling below the Mn–Cr closure temperature of ∼1,000°C (58). In this case, the ^53^Mn–^53^Cr age and cooling rate model can be used to estimate the time of impact injection of the metal component, provided that the initial temperature of the pallasites can be constrained. If the maximum temperature experienced was ∼1,700°C (20), the cooling rate at ∼1,300°C is ∼100°C Myr^–1^ (59), the Fe-Ni liquidus temperature is ∼1,500°C (59), and the Mn–Cr closure temperature is ∼1,000°C (58), the age of the impact can be constrained to between the time at which max temperature was reached and the point at which the temperature crosses the metal liquidus. The former happens at 2.60 Myr (Δt_CAI_) and the latter at 4.23 Myr (Figure S2, Supplementary Material). This cooling curve is not consistent with one calculated using low temperature fractional cooling rate information (see Supplementary Materials), however, which does not exceed the Fe-Ni liquidus temperature at any point. This suggests that the cooling history was more complex and indicates that without any further information on the interval between ∼1,300 and ∼700°C, the time of impact cannot be further constrained than between impactor differentiation (1.1–2.2 Myr Δt_CAI_) and the crossing of the Mn–Cr closure temperature of 1,000°C at 9.5 Myr (Δt_CAI_) (Figure S3, Supplementary Material). The timing of the impact and the cooling rate model are further discussed in the Supplementary Materials.

#### Broader significance

The main group pallasites are not the only pallasite group in the meteorite record. In addition, the pallasites include the Eagle Station group and a host of ungrouped meteorites including the pyroxene pallasites, Zinder, and Milton (20). The main group, along with most differentiated meteorites in the meteorite record, belong to an isotopic reservoir referred to as “non-carbonaceous” that is commonly attributed to having formed in the inner Solar System (3, 30). Contrastingly, the Eagle Station pallasite group belongs to a reservoir that is attributed to the outer Solar System, the “carbonaceous” reservoir (see 30), with Jupiter acting as a potential barrier to mixing between the 2 (3). The fact that pallasites appear to have formed both inside and outside of the orbit of Jupiter suggests that the process responsible for their formation may have been active over a large range in heliocentric distance, although it is possible that the Eagle Station pallasites were formed from an impact in the inner Solar System following or during gravitational scattering of bodies accreted in the outer, CC reservoir. The findings of this study, as well as previous studies invoking impact–injection mechanisms for pallasite formation (21, 22) imply that if impact–injection is the sole process responsible, it may have been Solar System-wide. Given that main group pallasites record very slow subsolidus cooling rates (5) that strongly suggest the impact injection did not disrupt the planetary body, they may be recording a stage of impact growth of differentiated bodies active throughout the entire Solar System. Considering that, at their simplest level, pallasites are mixtures of core and mantle material, it is possible that there are numerous processes that can lead to their formation. Therefore, a detailed investigation of oxygen isotope systematics between minerals within different pallasite groups is warranted to establish whether similar disequilibria are present.

## Materials and Methods

### Elemental concentrations

Elemental concentrations including Cr and Mn were measured on a ThermoScientific X-Series II quadrupole inductively coupled plasma–mass spectrometer calibrated against an in-house multi-elemental standard solution prior to column chemistry. Characterization of available pallasite slices and mineral phases was conducted prior to any isotopic analysis to provide petrological context. A Cameca SX100 Electron Probe Microanalyzer (EPMA) in the School of Environment, Earth, and Ecosystem Sciences at The Open University was used for quantitative major element characterization. The analyses were conducted using an acceleration voltage of 20 keV, a beam current of 20 nA, and beam diameter of 1 µm. The instrument precision is ± 0.02 wt% and the instrument calibration used crocoite as a Cr standard and was otherwise identical to that in (60). Crocoite was used in lieu of chromite as the latter standard was too degraded to provide reliable readings.

### Laser-assisted fluorination

The degree of precision required to identify very small differences in Δ^17^O necessitates the use of laser-assisted fluorination. The precision of this study is ± 0.09 ‰ for δ^17^O, ± 0.16 ‰ for δ^18^O, and ± 0.021 ‰ for Δ^17^O based on 52 analyses of an obsidian standard (see Supplementary Data). A detailed description of the instrument set up and procedure can be found in (61, 62). Laser-assisted fluorination is limited to bulk mineral analysis and therefore the isotope data are not spatially constrained. However, pallasites contain relatively coarse crystals of olivine and chromite, which negates the need for in situ analytical capability. The technique employs BrF_5_ as a fluorinating agent and a Photon Machines Inc CO_2_ IR laser to rapidly apply heat. The reaction with forsteritic olivine is (63)

(2) $$\begin{equation*}
M{g_2}Si{O_4} + 3Br{F_5} \to 2Mg{F_2} + Si{F_4} + 2{O_2} + 2Br{F_3} + 0.5B{r_2} + 0.5{F_2}.
\end{equation*}
$$

For chromite it is likely:

(3) $$\begin{equation*}
2FeC{r_2}{O_4} + 9Br{F_5} \to 2Fe{F_2} + 4Cr{F_3} + 4{O_2} + 9Br{F_3} + {F_2}.
\end{equation*}
$$

Obtaining a full reaction from chromite using BrF_5_ laser-assisted fluorination requires higher power density (∼10x) than that used for olivines as the chromite reaction can create greater quantities of fluorinated reaction products that remain in the sample well, obscuring yet to be reacted sample from both laser heating and BrF_5_.

Prior to analysis, samples were washed in 6 M HCl at 70°C for 3 minutes after (23, 26) in order to remove any terrestrial contamination and to facilitate a direct comparison of olivine results with the findings of (26). After washing, the samples were loaded into a Ni sample holder. The target weight for olivine was ∼1.8 mg and for chromite ∼1.2 mg. The lower weight is appropriate for chromite because too much sample material will generate large amounts of reaction products that can prevent full reaction of the sample. The amounts of O_2_ gas generated from chromite remain well in excess of that required to provide high precision results.

Following reaction, the sample gas was expanded along a gas clean up line containing two liquid N_2_ cryotraps and a heated bed of KBr (see 62). This ensured that waste gas was separated from the analyte O_2_ prior to its introduction into the mass spectrometer. Following the clean-up steps, the analyte gas was frozen on to 13X molecular sieve pellets cooled with liquid N_2_ prior to being expanded in to a ThermoFinnigan MAT 253 Dual Inlet isotope ratio mass spectrometer. Each run in the mass spectrometer was analyzed against a VSMOW-calibrated standard gas (O_2_-10) 10 times. The typical number of runs per sample was 6, meaning that each sample gas aliquot was subject to 60 comparisons to the standard gas unless otherwise stated in the results table. Each run was screened for ^14^NF_2_+ (m/z = 52), the fragment ion of NF_3_+, to check for potential interference by the ^14^NF+(m/z = 33) fragment ion on the ^16^O^17^O (m/z = 33) beam. In all cases, the amount of ^14^NF_2_ + detected was negligible. Further details on NF_2_ monitoring can be found in (64).

In addition to the olivines that were analyzed in conjunction with chromite samples, a larger sample group of 37 pallasite olivines were analyzed (Table S6, Supplementary Material), again following washing in 70°C HCl for 3 minutes as outlined in (23, 26). This was done in an attempt to replicate the bimodal Δ^17^O distribution of olivine reported by the latter study. These olivine samples are treated separately to those analyzed in conjunction with coexisting chromites to avoid confusion and those data are presented and discussed in the SI.

Oxygen isotope data are presented in the δ notation:

(4) $$\begin{equation*}
{\delta ^X}O \, \left( {\%_{\circ}} \right) = {10^3}\ \left( {\frac{{R_{sample}^X}}{{R_{standard}^X}} - 1} \right),
\end{equation*}
$$

where ${R^X}$ is the ratio of the isotope of interest over ^16^O. For linearized δ values this becomes:

(5) $$\begin{equation*}
{\delta ^{^{\prime}X}}O\ \left( {\%_{\circ}} \right)\ = {10^3}\ ln\ \left( {1 + \frac{{{\delta ^X}O}}{{{{10}^3}}}} \right).
\end{equation*}
$$

The Δ^17^O values reported are calculated by

(6) $$\begin{equation*}
{\Delta ^{17}}O{\rm{\ }}\left( {\%_{\circ}} \right){\rm{\ }} = {10^3}\ \ln \left( {1 + \frac{{{\delta ^{17}}O}}{{{{10}^3}}}} \right) - \lambda {10^3}\ln \left( {1 + \frac{{{\delta ^{18}}O}}{{{{10}^3}}}} \right).
\end{equation*}
$$

where $\ \lambda $ is the fractionation exponent (0.5262). In some studies, this is reported as Δ’ but is referred to without the prime symbol here following the convention of (65).

### Cr isotope measurements

The Cr isotope data presented in this study were collected using analytical methods modified from (66) and described in detail in (67) (see also Supplementary Information). Cr (and W) data are presented in the ε notation:

(7) $$\begin{equation*}
{\varepsilon ^X}Cr\left( {\%_{\circ}} \right) = {10^4}\ \left( {\frac{{R_{sample}^X}}{{R_{standard}^X}} - 1} \right),
\end{equation*}
$$

where ${R^X}$ is the ratio of the isotope of interest over ^52^Cr.

### Sample preparation, chemical purification, and W and Pt isotope measurements

Fukang and Seymchan were chosen for W isotope analyses because samples were readily available, and their metal portions exhibit an order of magnitude difference in Ir content; they therefore likely sample melt of different degrees of crystallization and could potentially reveal any relationship between differentiation age and metal melt evolution. A detailed description of the chemical purification of W and Pt as well as the analysis is provided in the supplementary material.

## Supplementary Material

pgac015_Supplemental_Files

## Data Availability

All data are included in the article and/or SI.
