# Supplementary material for: Isotopic evidence for pallasite formation by impact mixing of olivine and metal during the first 10 million years of the Solar System
Source: PNAS Nexus. 2022 Mar 9;1(1):pgac015. doi: 10.1093/pnasnexus/pgac015 (PMC9802258; doi:10.1093/pnasnexus/pgac015)
Supplement: pgac015_Supplemental_Files [file pgac015_supplemental_files.zip › PNASNEXUS-PNASNEXUS-2022-00134-T-s01.docx]

**Supplementary Information for**

Isotopic evidence for pallasite formation by impact mixing of olivine and metal during the first 10 million years of the Solar System

Richard J. Windmill*^1^, Ian A. Franchi^1^, Jan L. Hellmann^2^, Jonas M. Schneider^2^, Fridolin Spitzer^2^, Thorsten Kleine^2^, Richard C. Greenwood^1^, Mahesh Anand^1,3^.

*Richard J. Windmill

Email: [richard.windmill@open.ac.uk](mailto:richard.windmill@open.ac.uk)

**This PDF file includes:**

Supplementary text

Figures S1 to S5

Tables S1 to S6

SI References

Supplementary Information Text

Cr isotope method

Samples were dissolved in 2:1 HF:HNO_3_, dried and then re-dissolved in aqua regia to remove any remaining fluorides. For the chromite samples, digestion was done in a pressure bomb with 2 ml concentrated HNO_3_ for 96 hours at 196 °C. Following digestion, three stages of column chemistry was used to separate Cr in the olivine samples, only two stages were needed for the chromite samples.

**Stage 1 – Anion removal**

This stage was done primarily to remove Fe from the samples. One milliliter of AG1-X8 200-400 (10 ml Biorad®) resin was added to the columns and allowed to settle. The columns were then cleaned using 10 ml 3 M HNO_3_, followed by 18 ml of Milli-Q H_2_O (MQ), and lastly 12 ml 6 M HCl, after which they were each conditioned with 5 ml 6 M HCl. The samples were each loaded into 1 ml 6 M HCl, allowed to pass through the column, and were then collected. A further three stages of 6 M HCl was added in 1 ml steps and finally 6 M HCl in a 2 ml step was added. This was then collected with the sample. After this stage, the Cr samples were contained in 6 ml 6 M HCl in a vial with 7 ml volume. They were left overnight to dry down at 90 – 95 °C. Following drying down, concentrated HCl drops were added and allowed to dry down. In preparation for the second stage, 0.4 ml of 6 M HCl was added to each sample, the vials were closed and then placed on a hot plate at 120 °C for 12 hours. The last step involved adding 2 ml MQ prior to loading ensuring the samples were contained in 2.4 ml 1 M HCl.

**Stage 2 – Cr separation**

Fresh columns were prepared and 0.3 ml Biorad® resin was added. Initial cleaning steps included one addition of 16 ml 6 M HCl, followed by two of 16 ml MQ. The columns were then conditioned using 2 ml 1 M HCl and backwashed, then a further 2 ml 1 M HCl was added. The sample solution (2.4 ml 1 M HCl) was then loaded and the Cr cut collected using 3.6 ml 1 M HCl. The Cr cut was then contained in 6 ml 1 M HCl.

At this stage, the sample was checked for element abundances using a ThermoScientific XSeries II Quadrupole Inductively Coupled Plasma Mass Spectrometer. The olivine samples required Stage 2 to be repeated a further two times to remove Mg. The chromite samples were not subjected to Stage 3 as sufficient Cr was separated after Stage 2.

**Stage 3 – Cr clean up**

Heat shrink tube columns were prepared and 0.3 ml Biorad® resin was added. Initial cleaning was done with 9 ml 6 M HCl and two subsequent steps of 7 ml MQ, the latter MQ step was backwashed. The columns were then conditioned using two steps of 3 ml 0.5 M HNO_3_ and the sample was loaded in 3 ml 0.5 M HNO_3_. After the matrix cut had been taken, the final Cr cut was taken using 10 ml 2 M HCl so that the sample was contained in 10 ml 2M HCl. Organic removal was then performed using two steps of four drops of Aqua Regia that was twice dried down at 130 °C. Then, two drops of concentrated HNO_3_ and one drop of H_2_O_2_ were twice added and dried down at 80 °C. Finally, the sample was converted to HCl for the loading solution by adding two drops of concentrated HCl and being left to dry down at 120 °C, it was then taken up in 6 M HCl in order to create a 500-ppm loading solution and left to sit for an hour prior to loading on to the filament.

**Sample loading**

The sample solution (2 µl) was then added to a previously outgassed Re filament and heated at 0.7 A until dried. Then, 1 µl of 1000 ppm Al solution was added on top of the sample and left to dry at 0.7 A. After both had dried, 2 µl Si gel-H_3_Bo_3_ mixture was introduced and again dried at 0.7 A. When almost dry, a further 1 µl of the mixture was added to ensure coverage of the sample. Once the steps had dried, the current was increased to 1.2 A and the sample mixture allowed to homogenize for 5 seconds. The filaments with the sample mixture loaded were then loaded into the mounting wheel and placed in a ThermoScientific Triton Plus Thermal Ionization Mass Spectrometer (TIMS) prior to analysis.

**Corrections for cosmic ray exposure effects on Cr isotopes**

In addition to nucleosynthetic and radiogenic variations, Cr isotopes in meteorites may be affected by spallation of heavier elements (e.g., Fe and Ni) during prolonged exposure to galactic cosmic rays (GCR) (1). As such, cosmic ray effects are especially noticeable in samples with high Fe (or Ni)/Cr ratios (1), such as the olivines analyzed in this study. By contrast, chromites have low Fe/Cr ratios and their Cr isotope composition is, therefore, only minimally affected by cosmic ray-induced spallation. The spallation effects result in correlated ε^54^Cr-ε^53^Cr variations with a characteristic slope of 3.90 ± 0.03 (2). Thus, for co-genetic samples with a known common ε^54^Cr, cosmogenic ε^53^Cr variations can be quantified by back-projection of measured ε^53^Cr values to the pre-exposure ε^54^Cr of a sample. This approach could be used for the chromite-olivine pairs of this study, but only if chromite and olivine from each sample had the same unaltered Cr isotope composition. However, given that the low-Al-Mn chromite likely crystallized from the metal, and that this study seeks to test the hypothesis that silicate and metal portions have different planetary provenances, it would not be valid to use the chromite ε^54^Cr values as an initial composition for the olivine. To circumvent this problem, and because we have no independent method for obtaining a pre-exposure ε^54^Cr composition for the pallasite olivines, a pre-exposure composition that encompasses the inner Solar System values (i.e., the NC meteorites) was used as the initial value to facilitate the correction of ε^53^Cr values for the olivines. The values used for this range of inner Solar System compositions were the ureilites (3) and the average of enstatite chondrites (4), which are toward the lower and higher end of inner Solar System ε^54^Cr values, respectively. The average of these values was taken to give a broad NC average value. Following the GCR spallation correction, the ε^53^Cr values of the olivines are correlated with their Mn/Cr ratios, suggesting that the remaining variability in ε^53^Cr between different olivines is radiogenic in nature and, therefore, allows the time of Mn-Cr closure in these samples to be determined.

Cooling rate model

To help constrain the cooling rates and early evolution of main group pallasites, a simple exponential cooling rate was assumed:

|  | $T\left( t \right)=T_{0} e^{-\alpha t}$ | (1) |
| --- | --- | --- |

where $T$ is temperature, $t$ is time after CAIs (Myr), $T_{0}$ is initial temperature, and $\alpha=CR/T$ is the fractional cooling rate (5). The low-temperature metallographic cooling rates of Seymchan and Brenham range (including max errors) from 5.3 to 8.3 °C Myr^-1^ at ~640 °C on the basis of the metal containing <12 wt% Ni (6). The high-temperature cooling rate was likely 100-300 °C Myr^-1^ based on olivine zonation (7, 8) and the closure temperature of the Mn-Cr system in olivine is taken to be between ~950 and ~1050 °C (see Figure 5 in 9) based on measured average pallasite olivine grain sizes ranging from 1.24 to 6.21 mm (10). This constrains the closure temperature at 9.5 Myr (Δt_CAI_) to ~1000 °C and if $\alpha$ is taken from the low T cooling rate (cooling rate = 7.1 °C Myr^-1^ at 640 °C) and assuming exponential cooling, the minimum initial temperature $T_{0}$ is somewhere between ~1051-1162 °C. This $T_{0}$ seems very low given that liquidus temperatures for pallasite metal have been estimated at ~1500 °C (8) and suggests that the pallasites may have had a more complex early cooling history. Considering that the $\alpha$ value is calculated from low-temperature sub-solidus metallographic cooling rates in this case there is considerable scope for error given that the high-temperature cooling rates are unconstrained.

If the fractional cooling rate is instead taken from 100 °C Myr^-1^ at 1300 °C (*e.g.,* 8), the calculated cooling curve crosses the metal liquidus temperature, ~1500 °C, after 4.23 Myr (Figure S3), followed by the maximum temperature likely experienced, 1700 °C (11), at 2.60 Myr. Thus, for exponentially decreasing cooling to Mn-Cr closure at 1000 °C (*e.g.,* 9), a maximum temperature of 1700 °C (11), and a metal liquidus temperature of 1500 °C (8), the time of impact disruption can be constrained to between 2.60 and 4.23 Myr (Δt_CAI_). However, the lack of consistency between the cooling rate model with $\alpha$ calculated from the low temperature metallographic cooling values and that calculated with the high temperature values strongly suggests that the cooling history was more complex and that the fractional cooling rate varied over time. Furthermore, the exponential cooling curve calculated with $\alpha$ from the low temperature values (Condition set 1 on Figure S3) does not exceed the metal liquidus temperature at any point which indicates that the pallasites likely had a rapid initial cooling stage (as described by Condition set 2 on Figure S3) followed by slower cooling (as described by Condition set 1). This means that it is difficult to place tight constraints on the timing of impact and all that can be said with certainty is that the impact occurred after the differentiation of the impactor, 1.1-2.2 Myr Δt_CAI_ from Hf-W systematics, and prior to the crossing of the Mn-Cr closure at ~1000 °C (well below FeNi liquidus temperature) at 9.5 Myr (Δt_CAI_).

W isotope measurements and methods

Metal phases from Seymchan and Fukang were separated mechanically at The Open University by wrapping the sample in several layers of Teflon and gently tapping with a hardened steel hammer. Metal fractions (~1.5 g each) were digested in 32 ml HNO_3_–HCl–HF (10:5:1) at 120 °C on a hotplate for ~48 hours. After digestion, the samples were dried and re-dissolved in 60 ml 6 M HCl–0.06 M HF. Then, 3% aliquots were spiked with a ^180^Hf –^183^W tracer to precisely determine of Hf and W concentrations by isotope dilution (12, 13). Additionally, 12.5% aliquots (equivalent to ~200 mg solid sample) were taken for obtaining Pt isotopic compositions, which are required for correction of W isotope data for cosmic-ray exposure (CRE). The chemical separation of Pt followed the protocols described in (14), which is based on the techniques initially described by (15). In brief, Pt was separated from the sample matrix using an anion exchange chromatography step, where the samples were loaded in 1 M HCl–0.1% bromine water onto quartz glass columns and the Pt cut was collected in 14 ml 15.3 M HNO_3_ at the end of the elution sequence. Total yields of the Pt separation were typically ~85% and the total procedural blanks were negligible for all samples. Tungsten was separated from a remaining 87.5% solution aliquot of a sample using a two-step anion exchange chromatography as previously established for metallic samples (16).

The Pt and W isotope measurements were performed using a ThermoScientific Neptune Plus MC-ICP-MS at the Institut für Planetologie in Münster. Platinum was introduced into the mass spectrometer using a Savillex® C-Flow PFA nebulizer attached to a Cetac Aridus II desolvator. Total ion beam intensities of ~4×10−10 A were obtained for a 200 ppb Pt standard solution, an uptake rate of ~50 µL min^-1^, and a combination of standard Ni sample and (H) skimmer cones. Each isotope measurement consisted of 60 s baseline integrations (deflected beam) followed by 100 cycles of Pt isotope ratio measurements of 4.2 s each. Instrumental mass bias was corrected by internally normalizing to either ^196^Pt/^195^Pt = 0.7464 (denoted ‘6/5’), ^198^Pt/^195^Pt = 0.2145 (denoted ‘8/5’), using the exponential law. Possible interferences from Hg on ^196^Pt and ^198^Pt were monitored using interference-free ^200^Hg and were negligible, whereas isobaric interference on ^192^Pt from ^192^Os was corrected for by monitoring interference-free ^189^Os. Tailing effects on ^192^Pt and ^194^Pt from neighboring Ir isotopes have been corrected using the methods described (14).

Tungsten was introduced into the mass spectrometer using a Savillex C-flow nebulizer with an uptake rate of ~50 µL min^-1^ connected to a Cetac Aridus II desolvator. Using Jet sampler and X skimmer cones total ion beam intensities of ~2 × 10-10 A were obtained for a ~30 ppb W solution. Each measurement consisted of 60 s baseline integrations (deflected beam), followed by 200 isotope ratio measurements of 4.2 s each. Isobaric Os interferences on ^184^W and ^186^W were monitored on ^188^Os and were negligible for all samples. Instrumental mass bias was corrected relative ^186^W/^183^W = 1.98594 (denoted ‘6/3’) and ^186^W/^184^W = 0.92767 (denoted ‘6/4’) using the exponential law.

Both Pt and W data are given relative to the mean composition of Alfa Aesar solution standards that was analyzed bracketing the sample runs and are reported in the ε-notation (i.e., parts per 10^4^ deviations). For W, the reported ε^i^W represent the mean of pooled solution replicates (n = 5–7) with their corresponding 95% confidence intervals. For samples where Pt isotopes were analyzed several times, reported values represent the mean of pooled solution replicates. The accuracy and precision of the Pt isotope measurements were assessed by repeated analyses of the NIST 129c metal standard, which was doped with an Alfa Aesar solution standard containing highly siderophile elements (HSE). Reported uncertainties represent the long-term external reproducibility (2 s.d.) from previous studies at WWU (e.g., 17-19).

Pre-exposure ε^182^W values are calculated using the following equation (20):

|  | $\varepsilon^{182}W_{pre}=\varepsilon^{182}W_{meas}-{[\varepsilon}^{196}Pt_{meas}+ -0.006(\pm0.01)]*(-1.320\pm0.055)$ | (2) |
| --- | --- | --- |

Where the subscript $pre$ denotes pre-exposure, and the subscript $meas$ refers to measured.

Pt isotope methods

The chemical separation of Pt followed the protocols described in (14), which is based on the techniques initially described by (15). In brief, Pt was separated from the sample matrix using an anion exchange chromatography step, where the samples were loaded in 1M HCl – 0.1% bromine water onto quartz glass columns and the Pt cut was collected in 14 ml 15.3M HNO_3_ at the end of the elution sequence. Final Pt cuts were evaporated in concentrated HClO_4_ at 200°C several times to remove Os, which may generate an isobaric interference on ^192^Pt. This procedure resulted in pure Pt cuts with Os/Pt of ~2×10^-7^ to ~7×10^-6^ corresponding to <2 pg Os/measurement and resulting in interference corrections on ^192^Pt/^195^Pt of ~20 ppm for Seymchan, i.e., well within the external uncertainty of the measurements, and larger interference corrections of ~3.8 ε for Fukang. However, analyses of Pt solution standards with varying amounts of admixed Os showed that even Os interferences of this magnitude can accurately be corrected (14). In addition, only ε^196^Pt values are used for the quantification of CRE effects. Total yields of the Pt separation were typically ~85% and the total procedural blanks were negligible for all samples.

The Pt isotope measurements were performed on the ThermoScientific Neptune Plus MC-ICP-MS at the Institut für Planetologie. Instrumental mass bias was corrected by internally normalizing to either ^196^Pt/^195^Pt = 0.7464 (denoted “6/5”), ^198^Pt/^195^Pt = 0.2145 (denoted “8/5”), using the exponential law. Possible interferences from Hg on ^196^Pt and ^198^Pt were monitored using interference-free ^200^Hg and were negligible, whereas isobaric interference on ^192^Pt from ^192^Os was corrected for by monitoring interference-free ^189^Os (see above). Tailing effects on ^192^Pt and ^194^Pt from neighboring Ir isotopes have been corrected using the methods described in (14). The tailing corrections on ^192^Pt/^195^Pt were ~ 1 ε-unit for measurement solutions having Ir/Pt ≈ 1. Depending on the Ir/Pt of the sample and quality of the chemical separation of Ir from Pt, the Ir/Pt ratio of the measurement solutions were generally much smaller and varied between 0.03 to 0.27. Hence, the magnitude of the tailing corrections did not exceed the measurement uncertainty on ^192^Pt/^195^Pt (±1.2 ε, 2 s.d.). The Pt isotopic data are given relative to the mean composition of an Alfa Aesar solution standard that was analyzed bracketing the sample runs and are reported as ε-unit deviations. For samples analyzed several times, reported values represent the mean of pooled solution replicates. The accuracy and precision of the Pt isotope measurements were assessed by repeated analyses of the NIST 129c metal standard, which was doped with an Alfa Aesar solution standard containing highly siderophile elements (HSE). Reported uncertainties represent the long-term external reproducibility (2 standard deviation) from previous studies at WWU (e.g., 17-19).

Possible complicating oxygen isotope effects

When evaluating minor Δ^17^O differences between different minerals or attempting to resolve very small offsets between meteorite groups, it is crucial to use an appropriate value for the fractionation exponent ($\lambda$). Failure to do so can result in an apparent offset between different samples where none exists or, conversely, can make unrelated samples appear to fall on a single mass-dependent fractionation line. In the oxygen 3-isotope system, the fractionation exponent can vary over a small range between 0.5000 and 0.5305 (21-23) although in high temperature igneous environments this range is significantly reduced to ~0.525 – 0.529 (23, 24). Currently, there is no consensus on which $\lambda$ is appropriate for differentiated meteorite samples; this study adopts a slope of 0.5262 after (25). In addition to the planetary collision model treated in the main manuscript, other potential causes of the observed disequilibrium are evaluated here.

**Cosmic ray spallation**

Cosmic ray spallation of elements in minerals is known to affect a variety of isotopic systems (26, 27). Given that main group pallasites have long cosmic ray exposure (CRE) ages (28), the possibility that spallation could alter the isotopic composition of some constituent minerals must be considered. This is most easily achieved by comparing the Δ^17^O of olivine and chromite minerals in pallasites across a range of different CRE ages. Of the samples analyzed over the course of this study, four different pallasites have published CRE ages, from smallest to largest they are: Seymchan, Imilac, Brahin, and Brenham (see Table 15 in 28). Despite a ~130 Myr difference in the exposure ages of Seymchan and Brenham, there is no resolvable difference in Δ^17^O for either the olivine or the chromite in these meteorites or in those with intermediate CRE ages (see Tables 1 and 2 in main text). As a result of the lack of measured difference in Δ^17^O spanning the whole range of CRE ages for the meteorite group, cosmic ray spallation cannot be the cause of the Δ^17^O offset presented in this study.

**Anharmonicity**

It is possible that anharmonic effects may be the cause of the observed deviation from a mass-dependent fractionation line in low-Al-Mn subgroup minerals. Anharmonic effects have been invoked to explain mineral-specific Δ^17^O differences previously (e.g., 29), although the reported differences have subsequently been attributed to crustal contamination in the samples concerned (30). In addition, the fractionation of Li isotopes between aqueous solution and phyllosilicates has been shown to be influenced significantly by anharmonic effects (31). The nature and magnitude of any anharmonic contribution to mass-dependent fractionation results is an area of research that should be actively pursued as if anharmonic effects could give rise to a mineral-specific Δ^17^O offset similar to that presented in this study, the utility of oxygen isotopes as an indicator of planetary provenance in between differentiated meteorite groups close to the TFL would be severely undermined. Given that little is known about anharmonic effects and their contribution, they cannot be entirely discounted as a cause for the observed disequilibrium. However, considering the cooling rate (6) and palaeomagnetic evidence (32) that also hint at an impact origin for pallasites, it seems most likely that the observed Δ^17^O offset between low-Al-Mn chromite and olivine is caused by impact mixing. Finally, an investigation into the Δ^17^O contribution of a number of effects, including anharmonicity (and nuclear field shift effect), by 33 has shown that the magnitude is unlikely to be sufficient to generate Δ^17^O offsets observed in lunar and terrestrial samples (e.g., 23) on the order of 10 per meg. Given that these offsets are smaller than those reported here, it is unlikely that anharmonicity is the cause.

**Nuclear field shift effect**

A mechanism such as the nuclear field shift effect (NFSE) could potentially impart a mass-independent offset on otherwise mass-dependent fractionation. The nuclear field shift effect is known to impact fractionation in heavier isotope systems such as that of U but is thought to be too small to impact fractionation in light elements (34, 35). The NFSE causes nuclei with odd-numbered neutron counts to behave as though lighter than they are in chemical exchange reactions (36). Applied to oxygen isotopes, this would cause ^17^O to behave as though lighter and therefore any NFSE influence on mass-dependent fractionation in pallasite minerals should impart a shallowing of the slope in 3 isotope space, whereas the observed slope between low-Al-Mn chromite and corresponding olivine is steeper than possible through mass-dependent fractionation. Consequently, the NFSE cannot be the cause of the observed disequilibrium.

**Crossovers**

There are restricted cases where the $\lambda$ may vary infinitely for the three-oxygen isotope system. These “crossover” situations occur because the fractionation factors for different isotope ratios reach unity at different temperatures (37, 38). In this study, the fractionation factors of both isotope ratios for all pallasites are positive and so the conditions for a crossover situation are not met. Resultantly, this cannot be the cause of the disequilibrium.

**Multi-step fractionation**

A potential explanation for the Δ^17^O offset is the multi-step mass-dependent fractionation between the olivine, the chromite, and a third phase. This is possible given that slopes of mass fractionation can vary over a range of values and are broadly temperature dependent (e.g., 23, 39).

The possibility that multi-step fractionation is responsible for the observed disequilibrium is testable in a limited way using the data in this study. In addition, the olivine and chromite results, oxygen isotope data for a third mineral from the low-Al-Mn subgroup, farringtonite, are presented. A regression through the linearized low-Al-Mn subgroup olivine data with the farringtonite sample included yields a slope of 0.5114 ± 0.0152 (2 SE). This slope is considerably shallower than would typically be expected in high-temperature igneous processes (e.g., 23, 24) but is poorly constrained and is heavily dependent on a single farringtonite datum. If it is taken to be representative of farringtonite, it seems very unlikely that the low-Al-Mn subgroup olivine and farringtonite are in equilibrium given the high-temperature nature of their formation environment.

The low-Al-Mn chromite and farringtonite yield a slope in linearized 3-isotope space of 0.5285 ± 0.0060. Again, this slope is heavily dependent on the single farringtonite result, however it is consistent with expected high-temperature mass-dependent fractionation values. Assuming that the farringtonite is representative of low-Al-Mn subgroup farringtonite as a whole, it appears that the low-Al-Mn chromite and farringtonite plot on a single mass-dependent fractionation line and that the olivine is offset to less negative Δ^17^O values relative to that line. This is explainable if the chromite and farringtonite minerals are isotopically representative of the impactor; a conclusion that seems likely given that both minerals crystallized incorporating oxygen from the metal. The relationship between olivine, metal, and farringtonite may be described as follows (40-42):

|  | $3Mg_{2}SiO_{4}+4P+6Fe+8O_{2}=2Mg_{3}\left( PO_{4} \right)_{2}+3Fe_{2}SiO_{4}$ | (3) |
| --- | --- | --- |

This relationship would explain the reported abundance of farringtonite in samples containing lower Fo numbers (42) as well as the oxygen isotope results from this study.

Given that the low-Al-Mn chromite and the farringtonite likely incorporated metal-hosted oxygen during crystallization, as well as the fact that no realistic $\lambda$ can be used to link low-Al-Mn subgroup olivine and farringtonite, it seems likely that a multi-step mass-fractionation process is not responsible for the observed disequilibrium. Considering the poorly constrained farringtonite composition, however, such a mechanism cannot be completely discounted for the observed results.

An alternative possibility is that there is a fourth phase that was present that is not sampled and is in equilibrium with the analyzed minerals. Exploring this further, if this phase was in equilibrium with olivine and had a relationship defined by a $\lambda$ of 0.525, the lowest likely in high-temperature and igneous processes (23), then the intercept point of that slope with the chromite-farringtonite fractionation line would be at ~9 ‰ for δ^18^O, this equates to a fractionation of ~6 ‰ between the fourth phase and the low-Al-Mn subgroup olivine; this is extremely large in this context. Given the high-temperature environment of formation for pallasites, as well as the fact that the magnitude of fractionation should decrease with increasing temperature (see equation 2.55b in 43), it seems highly unlikely that such a large fractionation could occur. Pyroxene, a possible candidate for a missing fourth phase owing to its presence in some pallasites (e.g., 44), does not exhibit such a large fractionation relative to olivine at any temperature according to typical equilibration calculations (e.g., 45-47). If $\lambda$ values of 0.529 and 0.5305 are assumed for the olivine-missing phase relationship, the position of this fourth phase on the chromite-farringtonite fractionation line requires even more extreme and unlikely fractionation between it and the olivine. In the case of 0.529, a slope typical for high-temperature igneous processes (23, 24), the intercept would be at ~-28 ‰ for δ^18^O, requiring an enormous ~31 ‰ fractionation between the olivine and the fourth phase. For a slope of 0.5305, the high-temperature limit for oxygen, the intercept would be at ~-3.5 ‰ for δ18O, requiring a much less severe but still unrealistic ~7 ‰ fractionation. In addition to these fractionations, both the 0.529 and 0.5305 cases require the fourth phase to have a more negative δ^18^O than the olivine, a scenario we consider very unlikely considering that typically only oxide minerals exhibit this behavior during crystallization of a melt (48, 49) and at nothing like the magnitude required here. Because the range of high-temperature equilibrium mass-dependent$\lambda$ values span a range from ~0.525 – 0.529 (23, 24) the 0.525 slope seems the best candidate but as discussed, seems impossible for high-temperature fractionation.

Olivine homogeneity

In addition to the direct comparison of olivine and chromite presented in the main text, a set of 37 additional main group pallasite olivine samples were analyzed following HCl treatment as described in (50, 51). This was done with the aim of replicating the findings of the latter study, and to investigate the extent of any heterogeneous Δ^17^O distribution in olivines. Table S6 displays these results.

The 37 samples analyzed, from 18 different meteorites, yield unresolvable and completely homogenous Δ^17^O values at the precision afforded by this technique (-0.193 ± 0.018 ‰). There is some minor mass-dependent variability in δ^18^O and δ^17^O (Table S6), consistent with the smaller olivine dataset presented in the main text. These data show that, contrary to the findings of (51), the olivines display a high degree of homogeneity with respect to Δ^17^O and suggest that the mantle of the parent body was very well mixed and homogenized prior to olivine crystallization.

The cause for the discrepancy between this study and the study of (51) is unclear. It is possible that the Δ^17^O offset measured by (51) is the same as reported here between olivine and chromite, although we think this unlikely. If this were the case, it would require that all the olivine samples analyzed in this study happened to be in one of the two groups reported by (51) so as to appear homogenous. Given that we present data from both the high-Δ^17^O and low-Δ^17^O subgroup reported by (51) and see no offset, there would have to be a bimodal distribution of Δ^17^O in olivines within each pallasite to allow for us to select and analyze only one group. On the basis of the number of olivines analyzed in this study, as well as those of (52, 53), we think it highly unlikely that this is the cause of the disparity between the studies, especially considering that (51) found no evidence of the heterogeneous Δ^17^O between olivines within individual meteorites. A comparison of the data from the two studies is presented in Figure S4 with the Δ^17^O values from (51), (52), and (53) recalculated using the same $\lambda$ as in this study (0.5262). As alluded to for the olivine results presented in the main text, the olivine δ^18^O does show some isotopic heterogeneity in the set of 37 samples presented here. This δ^18^O variability [0.39 ‰ (2σ)] seems to be due to purely mass dependent sample heterogeneities, as evidenced by the homogeneity in Δ^17^O (see Table S6, Figure S4). Greenwood et al. (53) reported similar variability in δ^18^O of 0.24 ‰ (2σ) on a population of 24 pallasites and (51) reported 2σ variability in δ^18^O of 0.62 ‰ and 0.30 ‰ for their high Δ^17^O and low Δ^17^O subgroups, respectively. The variability in olivine δ^18^O may be caused by mass fractionation during the fractional crystallization of olivine from the precursor melt. Table S6 and Figure S5 detail average oxygen isotope results broken down by subgroups defined by (42). No subgroup-dependent variability in Δ^17^O is apparent.


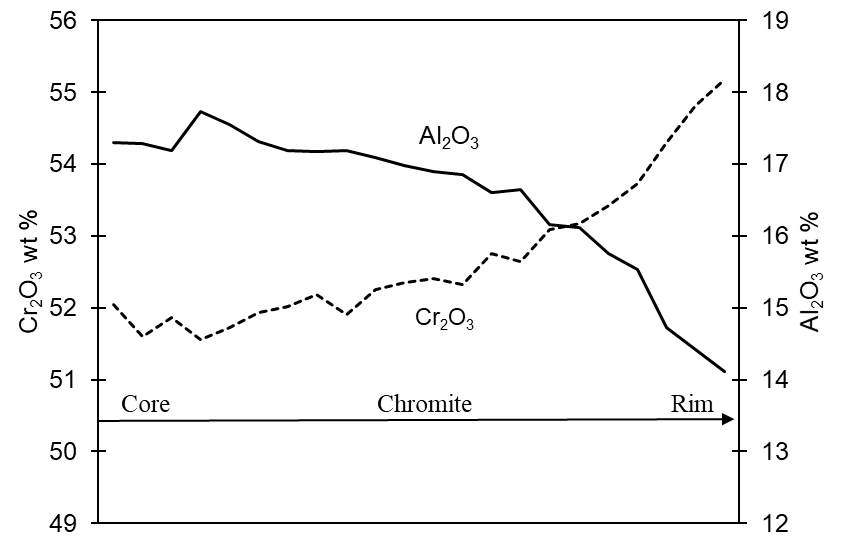


Fig. S1. A profile of Al2O3 vs Cr2O3 from core to rim (~1.5 mm) of Fukang chromite created using EPMA data.

| 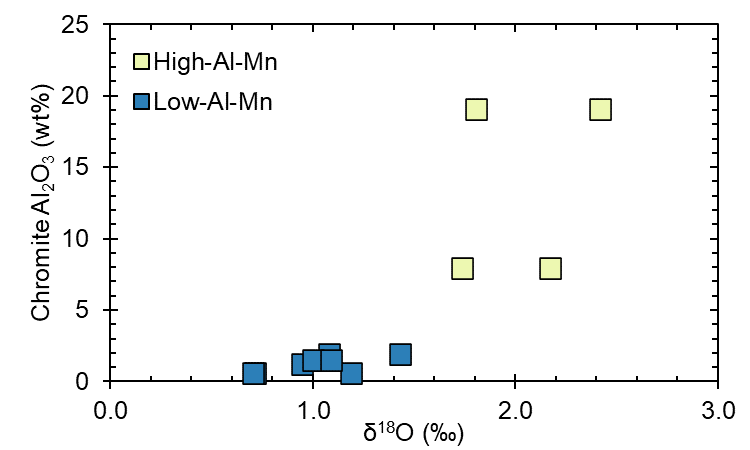  Fig. S2. A plot of chromite Al_2_O_3_ (wt%) vs δ^18^O (‰) for chromites. Hambleton is not shown as no compositional data is available, data for this plot are tabulated in Table S3. The Imilac Al_2_O_3_ is taken as the mean of the Ollague and Antofagasta data (Table S3). Note the clear difference in δ^18^O and Al_2_O_3_ between the two subgroups, it is unclear whether this is a bimodal relationship or whether these data sample parts of a continuum, further investigation is required to resolve this. |
| --- |


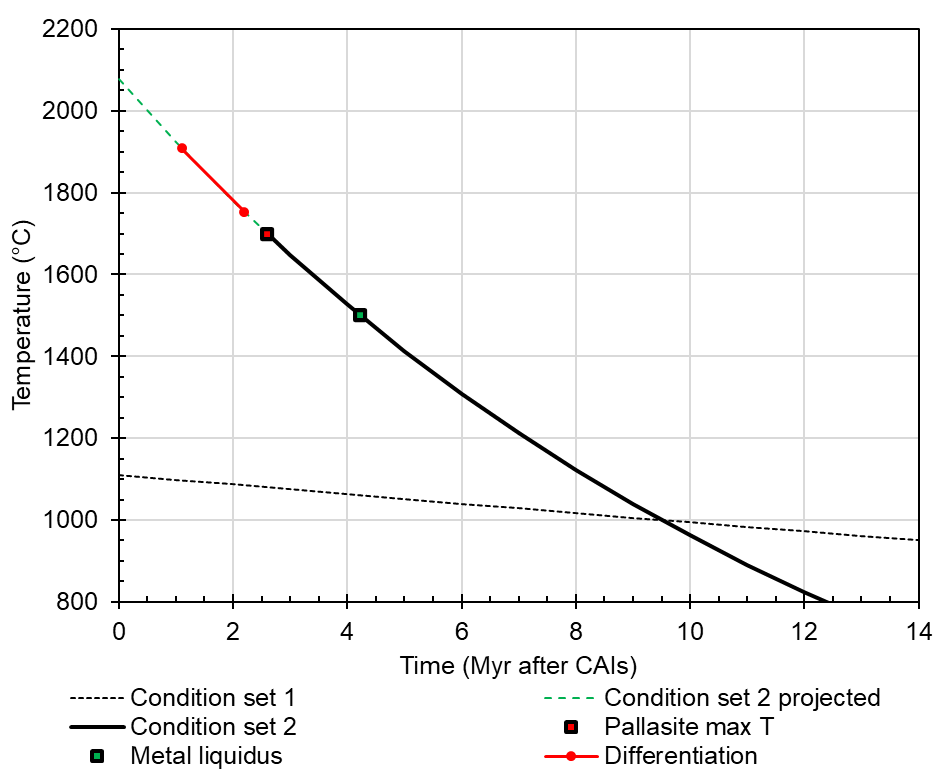


Fig. S3. Cooling rate models for the early history of the parent body. Condition set 1 are cooling rate 7.1 °C Myr-1 (average of Seymchan and Brenham in (6) at 640 °C, and Mn-Cr closure temperature of 1000 °C (9). Condition set 2 are, initial cooling rate of 100 °C Myr-1 at 1300 °C (lower bound of reference 8), and Mn-Cr closure temperature of 1000 °C (9). Condition set 2 crosses the max temperature of 1700 °C (11) at 2.60 Myr and the metal liquidus temperature of 1500 °C (8) at 4.23 Myr. Condition set 2 shows the projected temperatures prior to 2.60 Myr although this is unlikely to be accurate as discussed in the text. The range of differentiation ages (from W isotope chronology) are shown in red. Condition set 1 does not reach the metal liquidus temperature of 1500 °C (8).


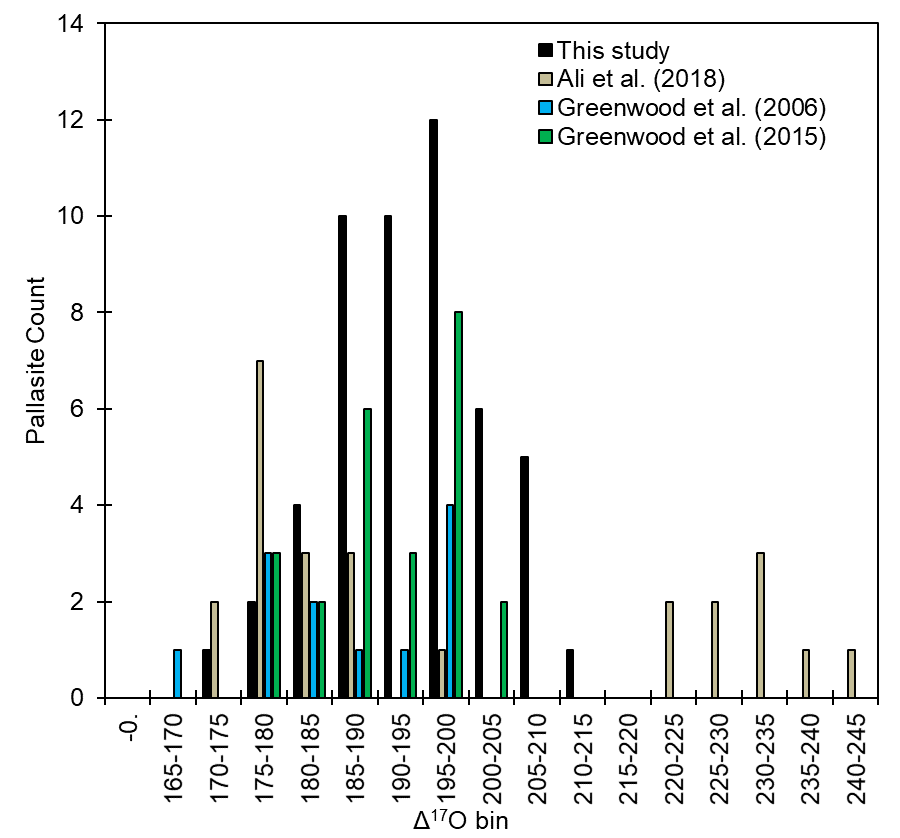
Fig. S4. A histogram showing the distribution of olivine Δ^17^O values across the reported range from four studies: this work, and references 51-53.

| 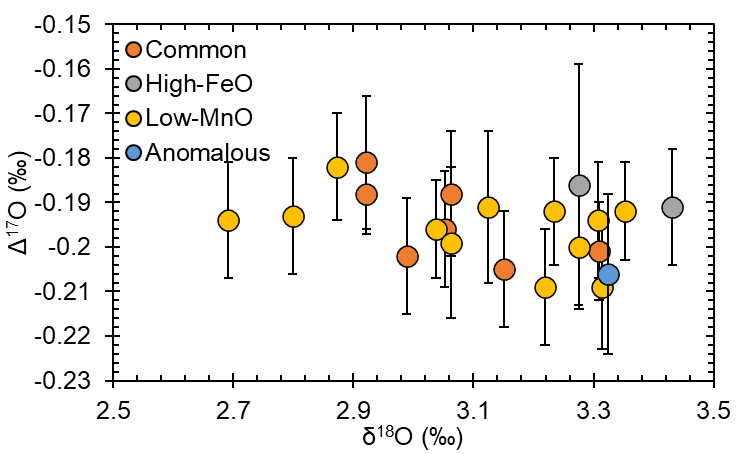  Fig. S5. Oxygen isotope results of olivines from Table S6 differentiated based on (42) subgroups, where known. The samples in Table S6 for which no subgroup is given by (42) are qualified with N/A and are not presented on this diagram. Note the lack of any difference in oxygen isotope results for the different subgroups. Seymchan (characterised as transitional by (42) is classed as Low-MnO here. |
| --- |

Table S1. Compositional data for analyzed olivines. O-M is olivine-metal rim, O-C is olivine-chromite rim, O is olivine core. The Fo numbers are calculated based on olivine core composition. The data are an average of n spot analyses. The abbreviation b.d. indicates below detection limit.

|  | Sericho | | | Seymchan | | | Fukang | | |
| --- | --- | --- | --- | --- | --- | --- | --- | --- | --- |
|  | O-M | O-C | O | O-M | O-C | O | O-M | O-C | O |
| n | 10 | 10 | 10 | 20 | 20 | 20 | 10 | 12 | 19 |
| Na_2_O | b.d. | b.d. | b.d. | b.d. | b.d. | b.d. | 0.07 | 0.03 | 0.02 |
| K_2_O | b.d. | b.d. | b.d. | b.d. | b.d. | b.d. | b.d. | b.d. | b.d. |
| MgO | 48.19 | 48.67 | 48.00 | 50.19 | 50.90 | 50.78 | 47.28 | 47.91 | 46.78 |
| CaO | b.d. | b.d. | b.d. | b.d. | b.d. | b.d. | b.d. | b.d. | 0.02 |
| MnO | 0.18 | 0.18 | 0.19 | 0.21 | 0.21 | 0.23 | 0.28 | 0.28 | 0.31 |
| FeO | 11.46 | 11.01 | 11.68 | 9.31 | 9.36 | 9.79 | 12.43 | 11.77 | 13.21 |
| Al_2_O_3_ | b.d. | b.d. | b.d. | b.d. | b.d. | b.d. | b.d. | b.d. | b.d. |
| Cr_2_O_3_ | 0.03 | 0.03 | 0.05 | b.d. | b.d. | 0.02 | b.d. | 0.20 | 0.04 |
| SiO_2_ | 39.95 | 40.21 | 40.34 | 39.33 | 38.02 | 38.84 | 39.91 | 40.33 | 40.19 |
| TiO_2_ | b.d. | b.d. | b.d. | b.d. | b.d. | b.d. | b.d. | b.d. | b.d. |
| Total | 99.84 | 100.14 | 100.27 | 99.08 | 98.54 | 99.68 | 100.00 | 100.53 | 100.58 |
| Cation proportions based on 4 oxygens | | | | | | | | | |
| Si | 0.989 | 0.996 | 0.999 | 0.974 | 0.942 | 0.962 | 0.988 | 0.999 | 0.995 |
| Ti | - | - | - | - | - | - | - | - | - |
| Al | - | - | - | - | - | - | - | - | - |
| Cr | 0.001 | 0.001 | 0.001 | - | - | - | - | 0.004 | 0.001 |
| Fe | 0.237 | 0.228 | 0.242 | 0.193 | 0.194 | 0.203 | 0.257 | 0.244 | 0.274 |
| Mn | 0.004 | 0.004 | 0.004 | 0.004 | 0.004 | 0.005 | 0.006 | 0.006 | 0.007 |
| Mg | 1.779 | 1.797 | 1.772 | 1.853 | 1.879 | 1.875 | 1.746 | 1.769 | 1.727 |
| Ca | - | - | - | - | - | - | - | - | 0.001 |
| Na | - | - | - | - | - | - | 0.003 | 0.001 | 0.001 |
| Total | 3.010 | 3.025 | 3.018 | 3.024 | 3.019 | 3.045 | 3.001 | 3.023 | 3.005 |
| Fo | 88.0 | | | 90.2 | | | 86.3 | | |

Table S2. Compositional data for the analyzed chromites. Bren. = Brenham, C-O is chromite-olivine rim, C-M is chromite-metal rim, C is chromite core. The data are an average of n spot analyses. The abbreviation b.d. indicates below detection limit.

|  | Bren. | Sericho | | | Seymchan | | | Fukang | | |
| --- | --- | --- | --- | --- | --- | --- | --- | --- | --- | --- |
|  | C | C-O | C-M | C | C-O | C-M | C | C-O | C-M | C |
| n | 30 | 10 | 10 | 20 | 10 | 20 | 20 | 15 | 15 | 20 |
| Na_2_O | b.d. | b.d. | b.d. | b.d. | b.d. | b.d. | b.d. | b.d. | b.d. | b.d. |
| K_2_O | b.d. | b.d. | b.d. | b.d. | b.d. | b.d. | b.d. | b.d. | b.d. | b.d. |
| MgO | 8.39 | 5.10 | 5.98 | 6.76 | 6.32 | 6.56 | 7.73 | 6.60 | 6.98 | 9.58 |
| CaO | b.d. | b.d. | b.d. | b.d. | b.d. | b.d. | b.d. | b.d. | b.d. | b.d. |
| MnO | 0.44 | 0.59 | 0.58 | 0.50 | 0.66 | 0.66 | 0.63 | 0.63 | 0.63 | 0.50 |
| FeO | 18.79 | 22.67 | 21.47 | 20.59 | 21.26 | 20.63 | 19.39 | 22.37 | 21.51 | 19.60 |
| Al_2_O_3_ | 0.61 | 1.44 | 1.11 | 1.93 | 1.39 | 1.39 | 1.51 | 10.98 | 8.55 | 19.09 |
| Cr_2_O_3_ | 71.97 | 72.02 | 72.79 | 71.59 | 70.12 | 71.57 | 70.01 | 57.86 | 61.56 | 49.65 |
| SiO_2_ | 0.13 | 0.10 | 0.09 | 0.11 | 0.07 | 0.11 | 0.11 | 0.07 | 0.07 | 0.08 |
| TiO_2_ | 0.06 | 0.03 | 0.02 | 0.07 | 0.05 | 0.06 | 0.08 | 0.26 | 0.17 | 0.43 |
| Total | 100.41 | 101.95 | 102.05 | 101.55 | 99.89 | 100.98 | 99.46 | 98.78 | 99.49 | 98.93 |
| Normalized cation proportions based on 4 oxygens | | | | | | | | | | |
| Si | 0.005 | 0.003 | 0.003 | 0.004 | 0.002 | 0.004 | 0.004 | 0.002 | 0.002 | 0.003 |
| Ti | 0.002 | 0.001 | 0.001 | 0.002 | 0.001 | 0.002 | 0.002 | 0.007 | 0.004 | 0.010 |
| Al | 0.025 | 0.059 | 0.045 | 0.079 | 0.058 | 0.057 | 0.062 | 0.441 | 0.344 | 0.724 |
| Cr | 1.976 | 1.990 | 2.000 | 1.957 | 1.958 | 1.975 | 1.940 | 1.559 | 1.662 | 1.263 |
| Fe | 0.546 | 0.663 | 0.624 | 0.595 | 0.628 | 0.602 | 0.568 | 0.637 | 0.614 | 0.527 |
| Mn | 0.013 | 0.017 | 0.017 | 0.015 | 0.020 | 0.020 | 0.019 | 0.018 | 0.018 | 0.014 |
| Mg | 0.434 | 0.266 | 0.310 | 0.349 | 0.333 | 0.341 | 0.404 | 0.335 | 0.355 | 0.459 |

Table S3. Compositional data for chromites relevant to this study including literature data. A table displaying the Al_2_O_3_, Cr_2_O_3_, and MgO for chromites in the pallasites analyzed for oxygen isotopes in this study. No compositional information is available for Hambleton. Oxygen data are averages of the samples analyzed in this study (Table 2, main text) with 2 standard deviations shown in brackets, the asterisk next to Brahin indicates it was a single sample and therefore no standard deviation is provided. The details column refers to the location in the chromite grain (if known): C=core, C-M rim=chromite-metal rim, C-O rim=chromite-olivine rim, C-T rim=chromite-troilite rim, C-St rim=chromite-stanfieldite rim, C-P rim=chromite-phosphate rim, C-S rim=chromite-schreibersite rim. The reference column details where the compositional information was obtained from. ^†^It is not known whether the Imilac samples analyzed were from the Ollague (O) or Antofagasta (A) masses (or other), but the oxygen data are displayed next to Ollague for simplicity.

| **Pallasite** | **Al_2_O_3_** | **Cr_2_O_3_** | **MgO** | **δ^18^O (2σ)** | **Subgroup** | **Details** | **Reference** |
| --- | --- | --- | --- | --- | --- | --- | --- |
| Sericho | 1.93 | 71.59 | 6.76 | 1.43 (0.49) | Low-Al-Mn | C | This study |
| Sericho | 1.11 | 72.79 | 5.98 |  | Low-Al-Mn | C-M rim | This study |
| Sericho | 1.44 | 72.02 | 5.10 |  | Low-Al-Mn | C-O rim | This study |
| Seymchan | 1.51 | 70.01 | 7.73 | 1.05 (0.13) | Low-Al-Mn | C | This study |
| Seymchan | 1.39 | 70.57 | 6.56 |  | Low-Al-Mn | C-M rim | This study |
| Seymchan | 1.39 | 70.12 | 6.32 |  | Low-Al-Mn | C-O rim | This study |
| Fukang | 19.09 | 49.65 | 9.58 | 2.11 (0.86) | High-Al-Mn | C | This study |
| Fukang | 8.55 | 61.56 | 6.98 |  | High-Al-Mn | C-M rim | This study |
| Fukang | 10.98 | 57.86 | 6.60 |  | High-Al-Mn | C-O rim | This study |
| Brenham | 0.61 | 71.97 | 8.39 | 0.87 (0.55) | Low-Al-Mn | C | This study |
| Brenham (met) | 0.59 | 70.10 | 9.20 |  | Low-Al-Mn | C | 54 |
| Brenham (met) | 0.19 | 70.76 | 8.30 |  | Low-Al-Mn | C-T rim | 54 |
| Brenham (met) | 0.13 | 70.80 | 7.53 |  | Low-Al-Mn | C-St rim | 54 |
| Brenham (chr) | 0.55 | 69.86 | 8.08 |  | Low-Al-Mn | C | 54 |
| Brenham (chr) | 0.23 | 69.03 | 5.75 |  | Low-Al-Mn | C-O rim | 54 |
| Brenham (chr) | 0.25 | 69.03 | 5.34 |  | Low-Al-Mn | C-T rim | 54 |
| Brenham (chr) | 0.26 | 68.88 | 5.18 |  | Low-Al-Mn | C-P rim | 54 |
| Brenham (chr) | 0.05 | 69.21 | 5.63 |  | Low-Al-Mn | C-M rim | 54 |
| Brenham (chr) | 0.05 | 69.42 | 6.55 |  | Low-Al-Mn | C-S rim | 54 |
| Brahin | 1.24 | 68.83 | 5.88 | 0.95* | Low-Al-Mn |  | 11 |
| Imilac (O) | 8.20 | 62.00 | 6.40 | 1.95 (0.61)^†^ | High-Al-Mn |  | 55 |
| Imilac (A) | 7.70 | 61.80 | 6.00 |  | High-Al-Mn |  | 55 |
| Hambleton |  |  |  | 1.11 (0.36) | Low-Al-Mn |  | N/A |

Table S4. W and Pt raw results for Seymchan and Fukang.

| Sample | | W (ppb) | | N | | ε^182^W (6/3) | | 95% conf | | ε^182^W (6/4) | | 95% conf | | ε^183^W (6/4) | | 95% conf | | ε^184^W (6/3) | | 95% conf | |  |  |
| --- | --- | --- | --- | --- | --- | --- | --- | --- | --- | --- | --- | --- | --- | --- | --- | --- | --- | --- | --- | --- | --- | --- | --- |
| Seymchan | | 309.9 | | 7 | | -3.32 | | 0.05 | | -3.42 | | 0.06 | | -0.07 | | 0.03 | | 0.05 | | 0.02 | |  |  |
| Fukang | | 184.4 | | 5 | | -3.75 | | 0.10 | | -3.60 | | 0.06 | | 0.12 | | 0.08 | | -0.08 | | 0.05 | |  |  |
|  | |  | |  | |  | |  | |  | |  | |  | |  | |  | |  | |  |  |
| Sample | N | | ε^192^Pt (6/5) | | 2 σ | | ε^194^Pt (6/5) | | 2 σ | | ε^198^Pt (6/5) | | 2 σ | | ε^192^Pt (8/5) | | 2 σ | | ε^194^Pt (8/5) | | 2 σ | ε^196^Pt (8/5) | 2σ |
| Seymchan | 3 | | 1.2 | | 1.2 | | 0.08 | | 0.18 | | -0.24 | | 0.20 | | 1.0 | | 1.1 | | 0.02 | | 0.15 | 0.08 | 0.07 |
| Fukang | 1 | | 0.9 | | 1.2 | | 0.10 | | 0.18 | | -0.38 | | 0.20 | | 0.7 | | 1.1 | | 0.02 | | 0.15 | 0.13 | 0.07 |

Table S5. Results of statistical testing of Δ^17^O disequilibrium for different subgroups. Low-Al-Mn and high-Al-Mn refer to which chromite subgroup the test between the olivine and chromite minerals was carried out. The 0.5262 and 0.5305 refer to the fractionation exponent used in the Δ^17^O calculation for the test. The p-values in bold are statistically significant at >99% confidence level. MC (Monte Carlo) refers to the t-test on 10^6^ simulated samples which was carried out in XLSTAT using the inbuilt function.

|  | High-Al-Mn 0.5262 | High-Al-Mn 0.5305 | Low-Al-Mn 0.5262 | Low-Al-Mn 0.5305 | Low-Al-Mn MC 10^6^ simulation |
| --- | --- | --- | --- | --- | --- |
| Difference | 0.013 | 0.008 | 0.021 | 0.011 | 0.011 |
| t (observed) | 3.202 | 1.908 | 6.222 | 3.374 | 3.374 |
| t (critical) | 2.120 | 2.120 | 2.074 | 2.074 | 2.088 |
| DF | 16 | 16 | 22 | 22 | 22 |
| p-value (two tailed) | **0.006** | 0.074 | **< 0.0001** | **0.003** | **0.003** |
| alpha | 0.05 | 0.05 | 0.05 | 0.05 | 0.05 |

Table S6. Oxygen isotope data for all olivine analyzed except those presented in the main text. The subgroup reference (42) allocated to each meteorite (where applicable) has been tabulated. The O wt% column is the calculated oxygen released from each sample based on sample gas pressure in the clean-up line. These olivine data are not considered when interpreting the olivine-chromite relationship. The errors are 2 standard error except for the errors on the averages (anomalous subgroup excepted) which are 2 standard deviation (underlined). The five rows at the bottom of the table are averages. The n column refers to the number of analyses of the sample gas for each sample against the reference gas.

| Pallasite | O wt% | δ^17^O | 2 SE | δ^18^O | 2 SE | Δ^17^O | 2 SE | Subgroup | n |
| --- | --- | --- | --- | --- | --- | --- | --- | --- | --- |
| *Acomita* | 42.6 | 1.337 | 0.015 | 2.894 | 0.006 | -0.185 | 0.014 | N/A | 60 |
| *Acomita* | 42.9 | 1.537 | 0.017 | 3.254 | 0.008 | -0.174 | 0.018 | N/A | 60 |
| *Brahin* | 40.8 | 1.279 | 0.013 | 2.799 | 0.007 | -0.193 | 0.014 | Low-MnO | 30 |
| *Brahin* | 42.4 | 1.221 | 0.013 | 2.692 | 0.006 | -0.194 | 0.013 | Low-MnO | 30 |
| *Brahin* | 43.0 | 1.484 | 0.013 | 3.219 | 0.007 | -0.209 | 0.013 | Low-MnO | 30 |
| *Brahin* | 44.5 | 1.533 | 0.015 | 3.314 | 0.010 | -0.209 | 0.014 | Low-MnO | 30 |
| *Brenham* | 43.6 | 1.545 | 0.014 | 3.308 | 0.011 | -0.194 | 0.014 | Low-MnO | 30 |
| *Brenham* | 46.1 | 1.523 | 0.014 | 3.276 | 0.006 | -0.200 | 0.014 | Low-MnO | 30 |
| *Esquel* | 43.9 | 1.370 | 0.012 | 2.989 | 0.007 | -0.202 | 0.013 | Common | 60 |
| *Finmarken* | 45.4 | 1.538 | 0.011 | 3.309 | 0.004 | -0.201 | 0.011 | Common | 60 |
| *Glorieta Mountain* | 42.1 | 1.423 | 0.014 | 3.063 | 0.006 | -0.188 | 0.014 | Common | 60 |
| *Glorieta Mountain* | 38.8 | 1.408 | 0.014 | 3.052 | 0.005 | -0.196 | 0.013 | Common | 60 |
| *Hambleton* | 44.7 | 1.329 | 0.012 | 2.873 | 0.005 | -0.182 | 0.012 | Low-MnO | 60 |
| *Hambleton* | 44.5 | 1.570 | 0.011 | 3.352 | 0.005 | -0.192 | 0.012 | Low-MnO | 40 |
| *Imilac* | 34.4 | 1.452 | 0.014 | 3.151 | 0.005 | -0.205 | 0.013 | Common | 60 |
| *Imilac* | 42.0 | 1.348 | 0.009 | 2.922 | 0.005 | -0.188 | 0.009 | Common | 200 |
| *Jepara* | 43.1 | 1.224 | 0.012 | 2.709 | 0.005 | -0.200 | 0.012 | N/A | 50 |
| *Lipovsky* | 47.0 | 1.371 | 0.010 | 2.984 | 0.008 | -0.198 | 0.010 | N/A | 60 |
| *Lipovsky* | 37.0 | 1.378 | 0.016 | 2.955 | 0.007 | -0.175 | 0.016 | N/A | 60 |
| *Marjalahti* | 42.6 | 1.356 | 0.016 | 2.922 | 0.008 | -0.181 | 0.015 | Common | 60 |
| *Mount Vernon* | 47.5 | 1.312 | 0.012 | 2.839 | 0.005 | -0.181 | 0.013 | N/A | 60 |
| *Mount Vernon* | 44.3 | 1.517 | 0.013 | 3.242 | 0.009 | -0.188 | 0.013 | N/A | 40 |
| *Pavlodar* | 44.6 | 1.541 | 0.018 | 3.324 | 0.009 | -0.206 | 0.018 | Anomalous | 60 |
| *Rawlinna* | 43.2 | 1.612 | 0.013 | 3.431 | 0.006 | -0.191 | 0.013 | High-FeO | 60 |
| *Rawlinna* | 43.2 | 1.536 | 0.026 | 3.275 | 0.008 | -0.186 | 0.027 | High-FeO | 30 |
| *Santa Rosalia* | 33.6 | 1.551 | 0.008 | 3.322 | 0.002 | -0.196 | 0.008 | N/A | 260 |
| *Santa Rosalia* | 45.9 | 1.503 | 0.014 | 3.199 | 0.005 | -0.178 | 0.014 | N/A | 60 |
| *Sericho* | 45.4 | 1.401 | 0.011 | 3.038 | 0.006 | -0.196 | 0.011 | Low-MnO | 60 |
| *Sericho* | 44.8 | 1.508 | 0.012 | 3.234 | 0.007 | -0.192 | 0.012 | Low-MnO | 40 |
| *Seymchan* | 44.9 | 1.411 | 0.016 | 3.063 | 0.009 | -0.199 | 0.018 | Low-MnO | 30 |
| *Seymchan* | 44.5 | 1.451 | 0.017 | 3.125 | 0.006 | -0.191 | 0.017 | Low-MnO | 50 |
| *Somervell Co.* | 41.5 | 1.550 | 0.01 | 3.318 | 0.009 | -0.195 | 0.014 | N/A | 30 |
| *Somervell Co.* | 42.0 | 1.373 | 0.015 | 3.008 | 0.007 | -0.208 | 0.015 | N/A | 30 |
| *Somervell Co.* | 43.8 | 1.411 | 0.014 | 3.039 | 0.007 | -0.187 | 0.014 | N/A | 30 |
| *Somervell Co.* | 39.7 | 1.356 | 0.017 | 2.938 | 0.008 | -0.188 | 0.016 | N/A | 30 |
| *Somervell Co.* | 43.0 | 1.433 | 0.012 | 3.107 | 0.010 | -0.200 | 0.011 | N/A | 30 |
| *Somervell Co.* | 44.5 | 1.557 | 0.014 | 3.317 | 0.014 | -0.187 | 0.016 | N/A | 30 |
| All | 42.9 | 1.439 | 0.203 | 3.104 | 0.391 | -0.193 | 0.018 |  |  |
| Low-MnO | 44.1 | 1.438 | 0.225 | 3.108 | 0.439 | -0.196 | 0.015 |  |  |
| Common | 41.3 | 1.414 | 0.133 | 3.058 | 0.275 | -0.194 | 0.018 |  |  |
| Anom. | 44.6 | 1.541 | 0.017 | 3.324 | 0.009 | -0.206 | 0.018 |  |  |
| High-FeO | 43.2 | 1.574 | 0.107 | 3.353 | 0.221 | -0.189 | 0.007 |  |  |

**SI References**

1. L. Qin, C. M. O’D. Alexander, R. W. Carlson, M. F. Horan, T. Yokoyama. Contributors to chromium isotope variation of meteorites. *Geochim Cosmochim Acta* 74: 1122-1145. (2010).
2. J. Liu, et al. Cosmogenic effects on chromium isotopes in meteorites. *Geochim Cosmochim Acta* 251: 73-86. (2019).
3. A. Yamakawa, K. Yamashita, A. Makishima, E. Nakamura. Chromium Isotope Systematics of Achondrites: Chronology and Isotopic Heterogeneity of the Inner Solar System Bodies. ApJ 720(1): 150-154. (2010).
4. A. Trinquier, J.-L. Birck, C. J. Allègre. Widespread 54Cr heterogeneity in the inner Solar System. ApJ 655: 1179-1185. (2007).
5. T. Kaiser, G. J. Wasserburg. The isotopic composition and concentration of Ag in iron meteorites and the origin of exotic silver. *Geochim Cosmochim Acta* 47, 43-58. (1983).
6. J. Yang, J. I. Goldstein, E. R. D. Scott. MG pallasites: Thermal history, relationship to IIIAB irons, and origin. Geochim Cosmochim Acta 74(15): 4471-4492. (2010).
7. S. J. McKibbin, T. R. Ireland, P. Holden, H. S. C. O’Neill, G. Mallmann. Rapid cooling of planetesimal core-mantle reaction zones from Mn-Cr isotopes in pallasites. *Geochem Perspect Lett*, 68-77. (2016).
8. P. H. Donohue, E. Hill, G. R. Huss. Experimentally determined subsolidus metal-olivine element partitioning with applications to pallasites. Geochim Cosmochim Acta 222: 305-318. (2018).
9. M. Ito, J. Ganguly. Diffusion kinetics of Cr in olivine and 53Mn-53Cr thermochronology of early solar system objects. Geochim Cosmochim Acta 70(3): 799-809. (2006).
10. G. F. D. Solferino, G. J. Golabek, F. Nimmo, M. W. Schmidt. Fast grain growth of olivine in liquid Fe-S and the formation of pallasites with rounded olivine grains. *Geochim Cosmochim Acta* 162: 259-275. (2015).
11. J. S. Boesenberg, J. S. Delaney, R. H. Hewins. A petrological and chemical re-examination of Main Group pallasite formation. Geochim Cosmochim Acta 89: 134-158. (2012).
12. T. Kleine, C. Münker, K. Mezger, H. Palme. Rapid accretion and early core formation on asteroids and the terrestrial planets from Hf-W chronometry. *Nature* 418(6901): 952-955. (2002).
13. T. Kleine, K. Mezger, C. Münker, H. Palme, A. Bischoff. 182Hf–182W isotope systematics of chondrites, eucrites, and Martian meteorites: chronology of core formation and mantle differentiation in Vesta and Mars. Geochim Cosmochim Acta 68: 2935–2946. (2004).
14. T. S. Kruijer, et al. Neutron capture on Pt isotopes in iron meteorites and the Hf-W chronology of core formation in planetesimals. *Earth Planet Sci Lett* 361: 162-172. (2013).
15. M. Rehkämper, A. Halliday. Development and application of new ion exchange techniques for the separation of platinum and other siderophile elements from geological samples. *Talanta* 44(4): 663-672. (1997).
16. J. L. Hellmann, T. S. Kruijer, J. A. Van Orman, K. Metzler, T. Kleine. Hf-W chronology of ordinary chondrites. *Geochim Cosmochim Acta* 258: 290-309. (2019).
17. T. S. Kruijer, et al. Protracted core formation and rapid accretion of protoplanets. Science 344, 6188(Jun): 1150-1153. (2014).
18. T. S. Kruijer, C. Burkhardt, G. Budde, T. Kleine. Age of Jupiter inferred from the distinct genetics and formation times of meteorites. Proc Natl Acad Sci USA 114(26): 6712-6716. (2017).
19. F. Spitzer, et al. Isotopic Evolution of the Inner Solar System Inferred from Molybdenum Isotopes in Meteorites. *Astrophys J Lett* 898: L2. (2020).
20. F. Spitzer, C. Burkhardt, F. Nimmo, T. Kleine. Nucleosynthetic Pt isotope anomalies and the Hf-W chronology of core formation in inner and outer solar system planetesimals. *Earth Planet Sci Lett* 576: 117211. (2021).
21. Y. Matsuhisa, J. R. Goldsmith, R. N. Clayton. Mechanisms of hydrothermal crystallization of quartz at 250°C and 15 kbar. Geochim Cosmochim Acta 42: 173-182. (1978).
22. E. D. Young, A. Galy, H. Nagahara. Kinetic and equilibrium mass-dependent isotope fractionation laws in nature and their geochemical and cosmochemical significance. Geochim Cosmochim Acta 66(6): 1095-1104. (2002).
23. E. D. Young, et al. Oxygen isotopic evidence for vigorous mixing during the Moon-forming giant impact. Science 351(6272): 493-496. (2016).
24. A. Pack, D. Herwartz. The triple oxygen isotope composition of the Earth mantle and understanding Δ17O variations in terrestrial rocks and minerals. Earth Planet Sci Lett 390: 138-145. (2014).
25. R. C. Greenwood, et al. Oxygen isotopic evidence for accretion of Earth’s water before a high-energy Moon-forming giant impact. *Science Advances* 4: 1-8. (2018).
26. H. Stauffer, M. Honda. Cosmic ray produced stable isotopes in iron meteorites. *J Geophys Res* 67: 3503-3512. (1962).
27. M. Honda, et al. Cosmogenic nuclides in the Brenham pallasite. *Meteorit Planet Sci* 37: 1711-1728. (2002).
28. G. F. Herzog, et al. Cosmic-ray exposure ages of pallasites. *Meteorit Planet Sci* 50(1): 86-111. (2015).
29. I. E. Kohl, P. H. Warren, E. A. Schauble, E. D. Young. Limitations on Δ’17O as a tracer of provenance revealed by mineral-specific values from lunar and terrestrial anorthosites. *Lunar Planet Sci Conf 48*, 2292. (2017).
30. E. J. Cano, Z. D. Sharp, C. K. Shearer. Distinct oxygen isotope compositions of the Earth and Moon. *Nat Geosci* 13\; 270-274. (2020).
31. R. Dupuis, M. Benoit, M. E. Tuckerman, M. Méheut. Importance of a Fully Anharmonic Treatment of Equilibrium Isotope Fractionation Properties of Dissolved Ionic Species as Evidenced by Li+ (aq). *Acc Chem Res* 50(7): 1597-1605. (2017).
32. J. A. Tarduno, et al. Evidence for a dynamo in the main group pallasite parent body. Science 338: 939-942. (2012).
33. E. A. Schauble, E. D. Young. Mass Dependence of Equilibrium Oxygen Isotope Fractionation in Carbonate, Nitrate, Oxide, Perchlorate, Phosphate, Silicate, and Sulfate Minerals. *Rev Mineral Geochem* 86, 137-178. (2021).
34. E. A. Schauble. Role of nuclear volume in driving equilibrium stable isotope fractionation of mercury, thallium, and other very heavy elements. *Geochim Cosmochim Acta* 71: 2170-2189. (2007).
35. S. Yang, Y. Liu. Nuclear field shift effects on stable isotope fractionation: a review. *Acta Geochimica* 35(3): 227-239. (2016).
36. J. Bigeleisen. Nuclear Size and Shape Effects in Chemical Reactions. Isotope Chemistry of the Heavy Elements. *JACS Au* 118(15): 3676-3680. (1996).
37. S. Skaron, M. Wolfsberg. Anomalies in the fractionation by chemical equilibrium of 18O/16O relative to 17O/16Oa. *J Chem Phys* 72: 6810. (1980).
38. P. Deines. A note on intra-elemental isotope effects and the interpretation of non-mass-dependent isotope variations. *Chem Geol* 199: 179-182. (2003).
39. Z. D. Sharp, J. A. G. Wostbrock, A. Pack. Mass-dependent triple oxygen isotope variations in terrestrial materials. *Geochem Perspect Lett* 7: 27-31. (2018).
40. E. Olsen, K. Fredriksson. Phosphates in iron and pallasite meteorites. *Geochim Cosmochim Acta* 30: 459-470. (1966).
41. Fuchs, L. H. Stanfieldite: A new phosphate mineral from stony-iron meteorites. *Science* 158: 910-911. (1967).
42. S. J. McKibbin, et al. Petrogenesis of main group pallasite meteorites based on relationships among texture, mineralogy, and geochemistry. Met Planet Sci 54(11): 2814-2844. (2019).
43. R. E. Criss. Principles of stable isotope distribution. Oxford University Press, Oxford, New York. (1999).
44. T. E. Bunch, D. I. Rumble, J. H. Wittke, A. J. Irving. Pyroxene-rich Pallasites Zinder and NWA 1911: Not Like the Others. *68th Annual Meeting of the Meteoritical Society*: 5219. (2005).
45. Y-F. Zheng. Calculation of oxygen isotope fractionation in anhydrous silicate minerals. *Geochim Cosmochim Acta* 57: 1079-1091. (1993).
46. Y. Bottinga, M. Javoy. Comments on oxygen isotope geothermometry. *Earth Planet Sci Lett* 20: 250-265. (1973).
47. Y. Bottinga, M. Javoy. Oxygen Isotope Partitioning Among the Minerals in Igneous and Metamorphic Rocks. *Reviews of Geophysics and Space Physics* 13(2): 401-418. (1975).
48. J. M. Eiler. Oxygen Isotope Variations of Basaltic Lavas and Upper Mantle Rocks. In Stable isotope geochemistry, *Rev Mineral Geochem* 43, Mineralogical Society of America, Washington D.C. (2001).
49. J. W. Valley. Oxygen isotopes in zircon. *Rev Mineral Geochem* 53: 343-385. (2003).
50. R. N. Clayton, T. K. Mayeda. Oxygen isotope studies of achondrites. Geochim Cosmochim Acta 60(11): 1999-2017. (1996).
51. A. Ali, et al. The oxygen isotope compositions of olivine in main group (MG) pallasites: New measurements by adopting an improved laser fluorination approach. Met Planet Sci 53(6): 1223-1237. (2018).
52. R. C. Greenwood, I. A. Franchi, A. Jambon, J. A. Barrat, T. H. Burbine. Oxygen Isotope Variation in Stony-Iron Meteorites. Science 313(Sep): 1763-1765. (2006).
53. R. C. Greenwood, et al. Geochemistry and oxygen isotope composition of MG pallasites and olivine-rich clasts in mesosiderites: Implications for the “Great Dunite Shortage” and HED-mesosiderite connection. Geochim Cosmochim Acta 169: 115-136. (2015).
54. J. T. Wasson, D. E. Lange, C. A. Francis, F. Ulff-Møller. Massive chromite in the Brenham pallasite and the fractionation of Cr during the crystallization of asteroidal cores. *Geochim Cosmochim Acta* 63: 7/8, 1219-1232. (1999).
55. T. E. Bunch, K. Kiel. Chromite and ilmenite in non-chondritic meteorites. *Am Min* 56: 146-157 (1971).
